# Chinese nutraceuticals and physical activity; their role in neurodegenerative tauopathies

**DOI:** 10.1186/s13020-020-00418-7

**Published:** 2021-01-06

**Authors:** Abdullahi Alausa, Sunday Ogundepo, Barakat Olaleke, Rofiat Adeyemi, Mercy Olatinwo, Aminat Ismail

**Affiliations:** 1 Department of Biochemistry, Faculty of Basic Medical Sciences, Ladoke Akintola University of Technology, Ogbomoso, Oyo Nigeria; 2 Department of Science Laboratory Technology, Faculty of Pure & Applied Sciences, Ladoke Akintola University of Technology, Ogbomoso, Oyo Nigeria

**Keywords:** Tau, Neurodegenerative diseases, Nutraceutical, Physical activity, Bioactive compounds

## Abstract

The onset of neurodegenerative disease has not only been a major cause of scientific worry, but of economic burden to the health system. This condition has been further attributed to mis-stability, deletion or mutation of tau protein, causing the onset of Corticobasal degeneration, Pick’s diseases, Progressive supranuclear palsy, Argyrophilic grains disease, Alzheimer’s diseases etc. as scientifically renowned. This is mainly related to dysregulation of translational machinery, upregulation of proinflammatory cytokines and inhibition of several essential cascades such as ERK signaling cascade, GSK3β, CREB, and PKA/PKB (Akt) signaling cascades that enhances protein processing, normal protein folding, cognitive function, and microtubule associated tau stability. Administration of some nutrients and/or bioactive compounds has a high tendency to impede tau mediated inflammation at neuronal level. Furthermore, prevention and neutralization of protein misfolding through modulation of microtubule tau stability and prevention of protein misfolding is by virtue few of the numerous beneficial effects of physical activity. Of utmost important in this study is the exploration of promising bioactivities of nutraceuticals found in china and the ameliorating potential of physical activity on tauopathies, while highlighting animal and in vitro studies that have been investigated for comprehensive understanding of its potential and an insight into the effects on human highly probable to tau mediated neurodegeneration.

## Introduction

At present, neurodegenerative diseases remain a great source of concern to public health, health practitioners and scientists at large, poising itself as a major economical headache to health care systems. Critical to the stability of microtubule it is a contaminant identified in 1975 by Weingarten and colleagues called TAU whose aggregates is a pathological indicator of tauopathies [1]. More so, tau has an overall incidence rate of 1.1 cases per 100,000 persons-year and a presumed onset of symptoms at 60 years and above. Psp recorded the highest incidence of tauopathy at a rate of [1.1 in men vs 0–6 in women], an inverse proportion to incidence recorded in CBS (0.1 in men and 0.3 women) [2].Conventional tauopathies, includes Alzheimer's disease (AD), corticobasal degeneration (CBD), progressive supranuclear palsy (PSP), Pick's disease (PiD), argyrophilic grain disease (AGD), Huntington’s disease (HD), and frontotemporal dementia with Parkinsonism-17 (FTDP-17), all of which are neuro-deteriorating diseases marked by the abnormal deposition of microtubule stability protein tau[3]. In 1975, the tau protein that has a prominent role in the assembly and stabilization of microtubules was first expressed in neurons within the central nervous system [4]. The indispensable role of tau in the transport of axons and neurite eruption cannot be overlooked [5]. Tauopathies such as Neurofibrillary tangles and formation of paired helical tangles (NFTs) can be traced to the withdrawal of tau from microtubules [6, 7], with tau mutations, mis splicing and abnormal post translational modifications being the risk factors [8, 9]. Although, tau induced neurodegeneration and mechanisms that leads to the upregulation of tau aggregation are yet to be clarified. AD is characterized by amyloid plaques made up of β-amyloid peptide and neurofibrillary tangles, comprising of hyperphosphorylated Microtubule Associated Protein Tau (MAPT or Tau), leading to loss of neurons and synapses as seen in other tauopathies. [10, 11]. Understanding the roles of these Aβ and tau pathologies is yet to be achieved after decades of research. Howbeit, difficulties in restating neurodegeneration via in vitro and in vivo animal models has led to a steady progress. Modulation of oxidative stress and inhibition of neuroinflammation to impede tau induced inflammation at the neuronal level and curb cognitive impairment should be considered towards the mitigation of in vivo oxidative process and aversion of neuronal damage. Thus, administration of nutrients and/or bioactive in combination with persistent exercise might aid the regulation of microtubule tau activity. Furthermore, neurobiological mechanisms (hormones, neurotropic factor levels, neurotransmitter secretion) can be triggered by exercise [12, 13], upregulating growth factors required for brain tissue development [14–16], regulating apoptosis and instigating neurogenesis in specific parts of the brain [17]. This study outlays the role of tau in brain diseases, the reassuring capacity of nutraceuticals found in china and exercise on tau mediated neurodegeneration, with the aim of creating the framework to further elucidate the interaction between these three facets and their effect on neurodegenerative diseases.

## Role of tau in brain diseases

**Fig. 1:**
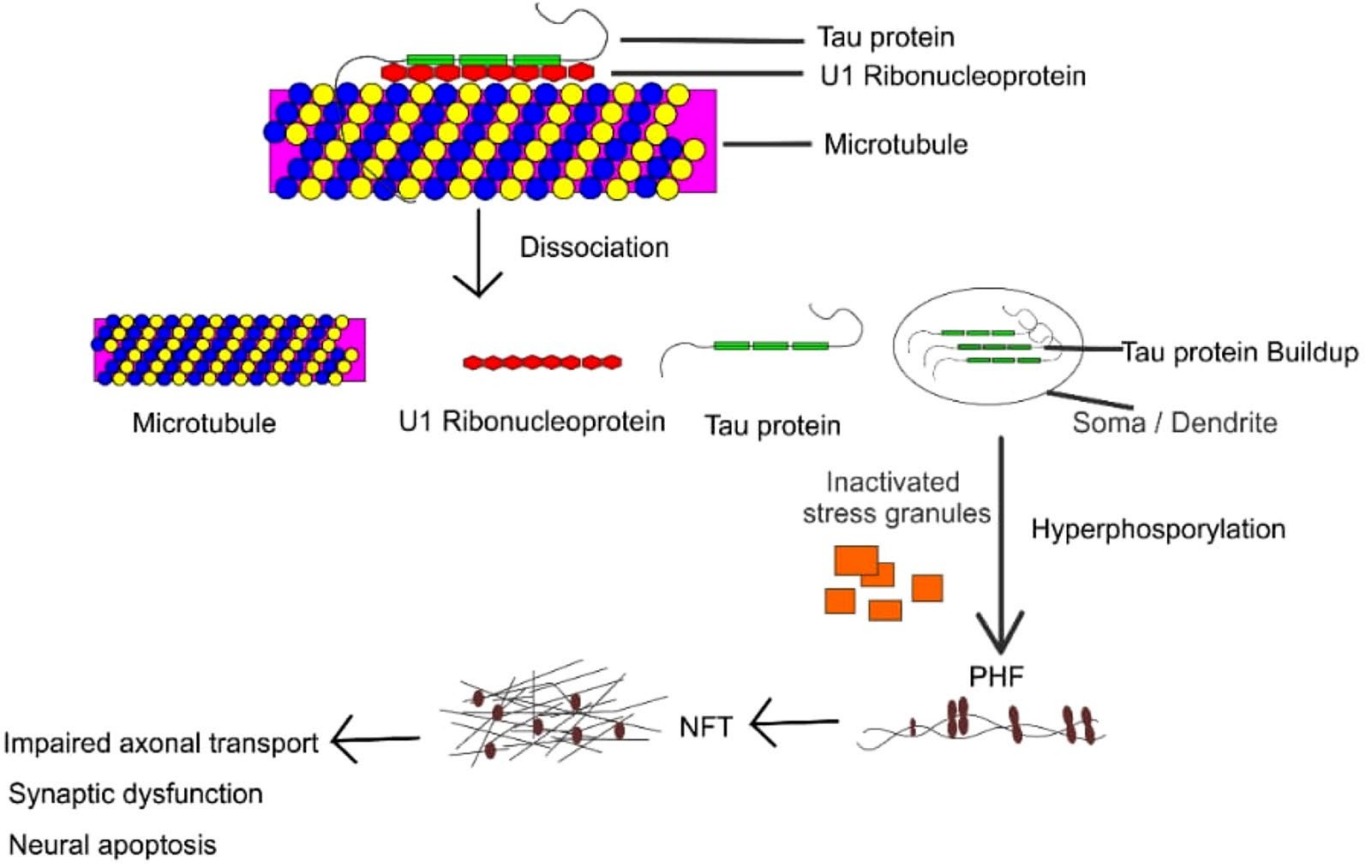
Schematic illustration of the role of tau in brain diseases

Microtubule associated protein tau encoded by tau gene (MAPT) is an intricate highly domain proteinous macromolecule associated with the axons of neurons enhancing stability of living species [4]. It is a multi-terminal protein, consisting of a highly acidic N-terminal and the neutral C-terminal connected by a central binding basic proline region [18]. Under pathogenic conditions, the multi domain tau builds up in the soma and dendrites of neurons. Strictly regulated by alternative splicing, tau exhibits six isoforms, distinguished by 2 N-terminal repeats (0 N, 1 N, or 2 N) and 3–4 microtubules binding repeats at the C-terminus (3R or 4R) [19]. Although tau plays important roles in the nervous system, uncoupling of microtubule from tau can be severe most significantly a leading cause of tauopathies [6, 7]. To maintain this relationship, tau protein is modulated by post-translational modifications and its isoforms by splicing is thus ensured. The action is thus possible by the downregulation of 3′ untranslated region of target mRNAs by microRNA (miRNA) [20]. Thus, inhibits the formation of truncated proteins and enhances microtubule-tau stability. With tau having a basic region which is responsible for its non-covalent interactions with the phosphate backbone of RNA [21]. Recent study further described the association of tau with RNA binding proteins U1 [22]. U1 are minute spliceosome nucleoproteins that binds to RNA highly essential in the regulation of RNA metabolism. However, the onset of tauopathies indicates manifestations such as dysregulation and aggregation of U1 ribonucleoprotein [23, 24], aggregation and improper hyper phosphorylation of insoluble tau [25], thereby leading to the detachment of tau from microtubules. Recent in vivo models further affirm that neurodegeneration and splicing defects occurs in U1-ribonucleoprotein tauopathies linked disease [26, 27]. Additionally, at the indication of translational stress, the cytoplasm assembles RNA and proteins to counteract these effects, thus leading to the formation of stress granules [27]. The activation of stress granules impairs proper translational machinery and cleaves RNA binding proteins which in response prevents the synthesis of truncated proteins. This action is possible due to the interaction of tau with proteins responsible for the formation of stress-granules. However, manifestation of brain diseases is characterized by the inactivation of this cascade [28, 29]. Furthermore, several studies have established the association of tau with ribosomes in regulating the translation machinery. This action is essential in maintaining the dendrites and synaptic plasticity of neurons [30–33]. Translational dysregulation has been associated with neurodegenerative diseases involving several complex molecular cascades that converges on the ribosome [34, 35]. Neurotic manifestation of tau-mediated neurodegeneration includes the onset of neurofibrillary tangles (NFT’s) as a result of aggregation of insoluble tau, although soluble oligomeric forms of tau have also been developed in neuronal dysfunction and physiological decay [36, 37]. However, the identification of human brain suffering from PSP, AD shows the assembly of oligomeric protein tau and similar brain function decline was observed in P301L mutant mice (rTg4510) [38–40] etc. Mice of familial FLTD-tau, results in the expression of frontotemporal dementia as observed in the articulation of human tau [37, 41]. The reduction in spine density, deterioration of permanent memory and changes in spine morphology was observed at the onset of AD during a mice study [42]. Similarly, the initiation of action potential by the specialized action initial segment and the regulation of neuronal excitability creating a barrier for the axonal compartment was inhibited in acetyl-mimic tau mice, resulting in the destabilization of AIS proteins [43]. Finally, post translational modifications of tau ensures proper stability of tau and microtubules, preventing the inactivation of stress granules. This extensive modification occurs via the action of several enzymes including kinase, acetylase, ubiquitin-degrading enzyme, methylase, glycosylase and protease enzyme [44]. However, aberrant post-translational modifications are critical in triggering synapse dysfunction and deterioration (Fig. 1).

### Neurodegenrative diseases associated to tau

Neurodegenerative disorder can be defined as the assembly of neurological issues which has impact on a subsection of neurons from a specific part of the focal sensory system and consistently induce their disintegration [45]. The positioning of harmful protein totals, indicate neurodegenerative disorder due to the misfolding and statement in the intra or extra neuronal locale [46]. Neurodegenerative Tauopathies integrate the compulsive state at which the microtubule stability protein tau, undergoes misfolding and gets reserved to shape the neurofibrillary tangles and tau fibers [7, 46]. Dysregulation of translational machinery could lead to a concomitant disassociation of tau protein and microtubules [34, 35]. Neurodegenerative diseases associated with tau (tauopathies) are classified into two, based on the pathological dispersal and the difference in biochemistry [47]. These basics classification namely primary and secondary tauopathies shares similar disease-relevant processes. Indicated by atrophy of the frontal cortex and temporal lobes, primary tauopathies are regularly in mix with decline subcortical mind zones, a member of the frontotemporal lobar degeneration (FTLD) diseases [48]. Existing isoforms of primary tauopathies includes 4R (AGD, PSP, CD, globular glial tauopathy), 3R (Picks disease) or 3R & 4R (Neurofibrillary tangle diseases) [49–51]. Additionally, the neurodegenerative AD is classified as a secondary 3R/4R isoform tauopathies [52].

### Progressive supranuclear palsy

PSP is a 4R isoform affiliated by tufted astrocytes, globose neurofibrillary tangles in grey matter and coiled bodies in oligodendrocytes in white matter [53]. It is an intricate clinicopathologic disease mostly affirmed by post mortem activities [54], with the mean age of onset is the mid-sixties and prevalence is estimated at 6 per 100,000 persons [1, 55, 56]. Affirmatively, the onset of ventrical supranuclear palsy and postural instability an indicator of PSP, while the onset of either signifies a clinically possible PSP [57]. Most commonly, PSP occurs with Richardson syndrome (PSP-RS), a nervous targeting disease with high probability of occurrence during autopsy [57–59]. Several existing occurrences of PSP includes, (PSP-PLS) [60, 61], (PSP-CBD) [62], PSP with progressive gait freezing [63, 64] and (PSP-C) [65, 66]. Environmental studies in France revealed that high rate of PSP emerges during the chronic exposure to heavy metals [67, 68]. Studies further revealed that extreme intake of electron transport chain complex inhibitor found in pawpaw [69, 70]. The occurrence of PSP is about 5–6 times increased in aberration or deletion of MAPT gene which is the major cause of PSP [71–73]. Several other indicators of PSP include increased oxidative stress, superior frontal cortex activation of lipid peroxidation markers, midbrain and subthalamic activation of lipid peroxidation markers, resulting in the activation of the inflammatory cytokines IL1β is observed in PSP [74]. To contain the damaging effects of PSP, the body defense system upregulates the synthesis and action of essential antioxidants majorly the superoxide dismutase, glutathione [75, 76]. Hyperphosphorylation remains a significant typical element of protein tau in PSP. This is due to the significant rise in reactive oxygen species synthesis, modification of kinase-based signaling cascades via enactment or outflow of the cascade components. In several tauopathies including PSP, increased activity of extracellular-regulated kinase 1 & 2 (ERK-1 /ERK-2), increased expression of stress activated protein kinase (SAPK/ JNK) and calcium/ calmodulin-dependent kinase II (CaM Kinase II) overexpression occurs [77], affirming the role of tau phosphorylation. Traditionally, protein kinase p38SAPK activated on the onset of stress is linked with reactive oxygen species induced oxidative stress. Several clinical trial drugs examined for PSP includes valproic acid (NCT00385710, NCT00703677), tideglusib underwent a double-blinded placebo trial which also failed in PSP treatment. Others include salsalate (NCT02422485), CDK5 (NCT04253132) [78] etc.

### Corticobasal degeneration

Corticobasal degeneration (CBD), is a member of the 4R tauopathies basically associated with behavioral, cognitive and motor disorder [79]. It was formerly referred to as a corticodentatonigral degeneration, closely associated with neuronal achromasia [80]. While a clear and definite figure on the prevalence of CBD is currently unavailable, an average of 4.9–7.3 cases in every hundred thousand persons was accounted by eastern European and Asian population study [81]. More so, despite the naming of this condition CBD since 1989, few researchers preferably name it as corticobasal ganglionic degeneration [82–84]. Notably, is the clear differentiation of CBD from corticobasal syndrome (CBS). CBS is clearly a CBD phenotype manifested by the emergence of two or more crooked cortical ignitions such as myoclonus, limb apraxia and parkinsonism [85]. Over expression of the kinase enzyme (responsible for phosphorylation), causes an improper hyperphosphorylation of insoluble tau and a concomitant release of tau protein from microtubule in the brainstems, somatosensory region, basal ganglia, and supplementary motor cortices as the major manifestation of CBD [86]. Thus, causing a loss of microtubule function. Dementia ranging from FTD-AD prototype, CBS, PSP, RD are the frequently occurring CBD phenotype [87]. Pathologically, the trademark injury of CBD in most cases is astrocytic plaque, caused by the deposition of abnormal tau, abnormal proteins in AD, Parkinson disease and Multiple sclerosis (MS) [88], cohabiting normally with dystrophic neurites and irregularities in discharge of tau muddles by the oligodendroglia cytoplasm [89, 90]. Furthermore, it is characterized by the invasion of neurotransmitters by prion like proteins [91], thus sharing an overlapping clinical and pathological feature with PSP. This effect leads in the expression of proinflammatory interleukin 1beta (1L1β), interleukin 6 (IL6), and increasing level of tumor necrosis factor alpha (TNFα), causing plaque and microglia degeneration, a hallmark of neuroinflammation [92]. However, despite the relation of CBD with PSP, biochemical features of both 4R tauopathies differs. Most notably, is a double 37kda band tau fragment as compared to the single 33kda band fragment of PSP [88, 93]. While it is clear that mutation of the encoding MAPT gene, situated on 17q21.31 chromosome is the hallmark of CBD emergence, postmortem examination of CBD patients observed a connection between single nucleotide polymorphisms (SNPs) in MAPT H1 haplotype and the hydrolyzing enzyme Rab GTpase, acting on myelin associated oligodendrocyte basic proteins (MOBP), essential in the effective functioning of myelin sheath [94]. Currently, there still remains no globally accepted therapy for the treatment of CBD, suspected patients may progress from acute to chronic, requiring intensive care, expressing behavioral and cognitive disorders [95]. However, several drugs have been tried in the management of CBD which includes levodopa, benzodiazepines [96], levetiracetam [97], intramuscular administration of botulinum toxin [98]. As science grows each day, the concept of CBD becomes clearer, although autopsy is still the major diagnosis of the 4R tauopathy CBD at present.

### Argyrophilic grain disease

Argyrophilic grain (ArG) is a 25 nm smooth tubules and straight filament, spindle like in shape or a spherical lesion in appearance, identified majorly at the onset of Argyrophilic grain disease (AgD). It is responsive to silver iodide staining techniques, and visible in abnormally phosphorylated tau proteins, for diagnosis purpose [99]. ArG is distributed across trans entorhinal, entorhinal cortices, sub nuclei of amygdala, amygdaloid complex, and in minute cases the basal portions of claustrum [100, 101]. In variant to NFT, ArG are absent in neuronal cell perikaryal [102]. A notable hallmark of AgD is the appearance of coiled bodies, mostly branched oligodendroglia inclusions around the nucleus [103, 104]. They are located around the cortices and subcortices of ArG at the manifestation of AgD, distinguished by hyperphosphorylation, similar to pentangle neurons. More so, the prevalence of AgD increases in degenerative dementia cases, and also directly proportional to age [105]. Frontotemporal cortical atrophy is also observed upon gross examination [106]. Normal phosphorylation of microtubule tau transpires at Serine (202,214,235,396,404,422) and Threonine (181,205, 231) although excessive kinase activity of tau protein occurs at Ser 262 [107]. These residues are catalyzed by mitogen-activated protein kinase, SAPK/JNK, glycogen synthase kinase 3β (GSK-3β) & p38 kinases [108]. AgD remains a member of the 4R tauopathies, however immunohistochemistry remains the major diagnosis tool, in detailing the pathologic conditions of an AgD patient.

### Alzheimers diseases

AD is one of the leading causes of medical challenges, affecting mankind and derailing healthy living. The challenge to contain and improve the management of AD has been on over years since it was coined in 1970. A major pathophysiological protein, relevant to the progression of AD is the microtubule associated tau protein. Present evidences point at the aggregation of abnormal tau at the synapse and nucleus, elucidating a loss of tau function as the pathological mechanism of AD emergence [109–111]. Distinctive features at the hippocampal and temporal cortical regions, identified by the deposition of insoluble hyperphosphorylation tau, NFT, and deposition of β-amyloid (Neurotic plaques) are indicative features of AD. This proceeds to several worrisome conditions such as memory loss, impaired behavioral activities, visuospatial function impairment and loss of cognitive functions [112, 113]. Similarly, lessening in Aβ 42, low proportion of Aβ 42 to amyloid β 1–40 proportions, expansion of t-tau and p-tau, incorporation of amyloid markers (Aβx-38, Aβx-40, Aβx-42 and solvent antecedent protein), complex fiber axonal degeneration, and neuroinflammation indicating protein (chitinase-3-like protein1/ YKL-40) are all molecular signatures of AD [114, 115]. Pathologically, four in every five AD patients expresses the deposition of alpha-synuclein in lewy body dementia [116], and about three in every five patients with AD displays lewy-body type syncleinopathy [117]. While 4R tauopathies are histologically indicated in the pathology of AD [11], deposition of TDP-43 deposits, linked to β-amyloid dependent and independent cascades are also recognized in AD brains [118, 119]. Finally, there are several G-protein coupled receptors (GPCR), mediating the phosphorylation of tau protein via kinases majorly via the ERKs, GSK-3β, protein kinases and CDK-5 [120, 121]. Activation of GPCR by either of its activation cascade notably intracellular activation, transactivation, classical activation, diphasic activation, or biased activation cascade, allows for the protection against cell degeneration, oxidative and cytosolic stress protection [122–124]. However, the progression of AD is characterized by imbalances between GPCR-mediated kinases, thus leading to improper hyperphosphorylation of tau-microtubules [128, 129] (Table 1).

**Table 1:** Summary of the pathophysiology, disease indicators, current treatment strategies and drugs used in neurodegenerative tauopathies

Tauopathies	Patophysiology	Disease indicator	Current treatment strategies	Drugs
Alzehimer’s diseases	Characterized by a defined pattern of tangles from the trans-entorhinal cortex → entorhinal cortex → CA1 region of hippocampus and finally to the frontal, temporal, and parietal cortices, thus leading to neuronal loss and atrophy which induces inflammation and amyloid plaques deposition [125]	Formation of senile plaques (SP) [126]	Amyloid-β-peptide vaccination Secretase inhibitors Anti-inflammatory agents Cholesterol reducing drugs [127]	Donepezil Galantamine Memantine Rivastigmine [128]
Progressive supranuclear palsy	Characterized by development of NFT in astrocytes with the diffusion of plaques and lewy bodies in the brainstem cell nuclei & cerebral cortex. However, in long standing cases, the purkinje cells of the cerebellum are affected leading to the dilation of the third & fourth ventricles [129, 130]	Gait difficulty and falls Dystonia and personality change Non-specific dizziness Palilalia Compulsive spitting [131]	Drug therapy Electroconvulsive therapy [PSP]	Levodopa Dopamine agonists Pergolide Physostigmine Zolpidem Benztropine [PSP]
Corticobasal degenration	Characterized by asymmetric frontoparietal cortical atrophy and asymmetric hyperexcitability of the motor cortex, thus leads to the progression of astrocytic plaques	Ataxic gait Myoclonus Dystonia Dysphagia Dysarthria	Imaging techniques such as computerized tonography scanning (CTS) & Magnetic Resonance Imaging (MRI) Clinical testing and work up (Electroencephalogram; EEG)	Baclofen Botulinum toxin Clonazepam Benzodiazepines
Argyrophilic disease	At the anterior entorhinal cortex, there is mild involvement of the cortical and basolateral nuclei of the amygdala. There is also mild involvement of mammary bodies, nucleus accubens and rare grains in the mid brain, thus leading to impaired UPS function. [132]	Episodic memory loss Cognitive decline and dementia Behavioral abnormalities Emotional imbalances [133, 134]	Anti-phosphorylation strategies Anti-aggregation strategies	Rhodamines Thiacarbocyanines N-phenylalanimes Anthraquinones Levodopa

## Role of chinese nutraceuticals in tau neurodegenrative diseases

Emerging from the credible combination of nutrient and pharmaceuticals background is the word termed called nutraceuticals [135]. Also referred to as bioceuticals, they are classified as dietary supplements and food additives by FDA, highly efficient in protecting health and maintaining diseases and as such increase’s life expectancy [136, 137]. Statistical evaluation of dietary supplements consumption revealed that 68% [138], 83–86% [139] and about 63–70% consumption rate across United states, Germany, Italy and Australia respectively [139–141]. General to tau neurodegenerative disease is the synthesis of truncated proteins as observed in Aβ in AD, misfolded tau & TDP-43 in TBI, and misfolded Aβ & tau in other tauopathies [142, 143]. These concomitantly leads to the upregulation of detrimental molecular cascades that enhances degeneration. Neurodegeneration occurs when the misfolded proteins activates destructive molecules, most notably free radicals, mitochondrial DNA damage, oxidative stress, iNOS and COX-2 by the overexpression of inflammatory cytokines via the NF-Kβ cascade induction [144, 145]. On the other hand, CREB, ERK signaling cascade, GSK3β [146], and PKA/PKB (Akt) signaling cascades known to promote protein processing, normal protein folding, cognitive function, and microtubule associated tau stability becomes inhibited [145, 147, 148]. However, nutraceuticals are effective in the management of tauopathies. They act by upregulating ERK, Akt, GSK3β and CREB cascades, along with the exertion of anti-inflammatory, and reactive oxygen species ameliorating capabilities. Thus, preventing oxidative stress and aid cognitive functioning. This study further explores some nutraceuticals found or produced in China and their role in management of tauopathies.

### Garlic (*Allium sativum*)

**Scheme 1:**
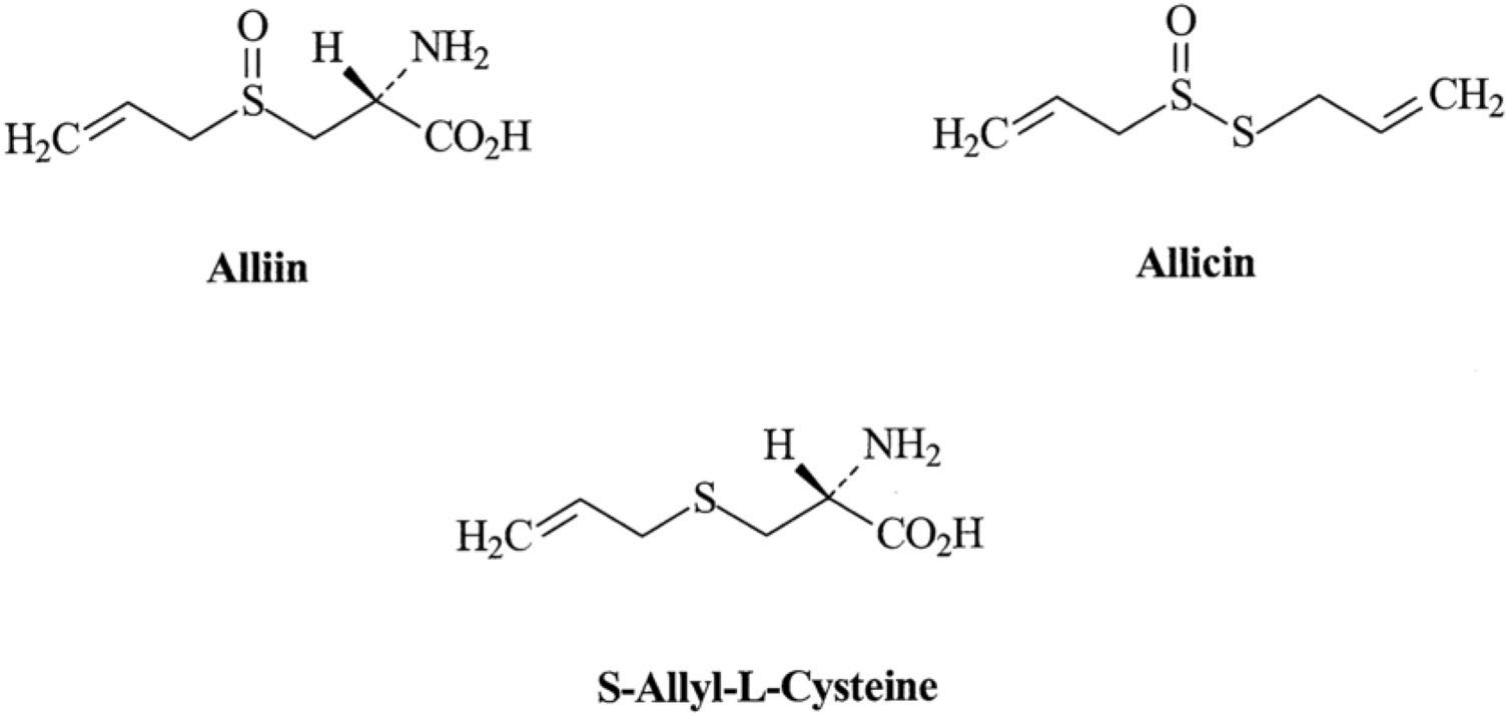
Structures of bioactive compounds present in *Allium sativum*

Belonging to the family of Alliaceae, it is a highly cultivated vegetable believed to originate from central Asia in the past 6000 years ago and used in Chinese medicines in the past 3000 years ago [149]. It is an abundant organo-sulfurous compound, efficient in management of several cardiovascular diseases. Their sulfur compound exists as diallyl sulfide, s-allyl-l-cysteine sulfoxides (Allin), S-allyl-l-cysteine and allicin (diallyl thiosulphate), all generally referred to as Allium compounds [150, 151]. Although containing substantial number of macromolecules, (28% CHO, 3.2% protein, 1.5% fibers and 6.5% water), diallylsulphate; an organosulfur compound was studied to be responsible for its pharmacological activity [152, 153] (Scheme 1).

**Fig. 2:**
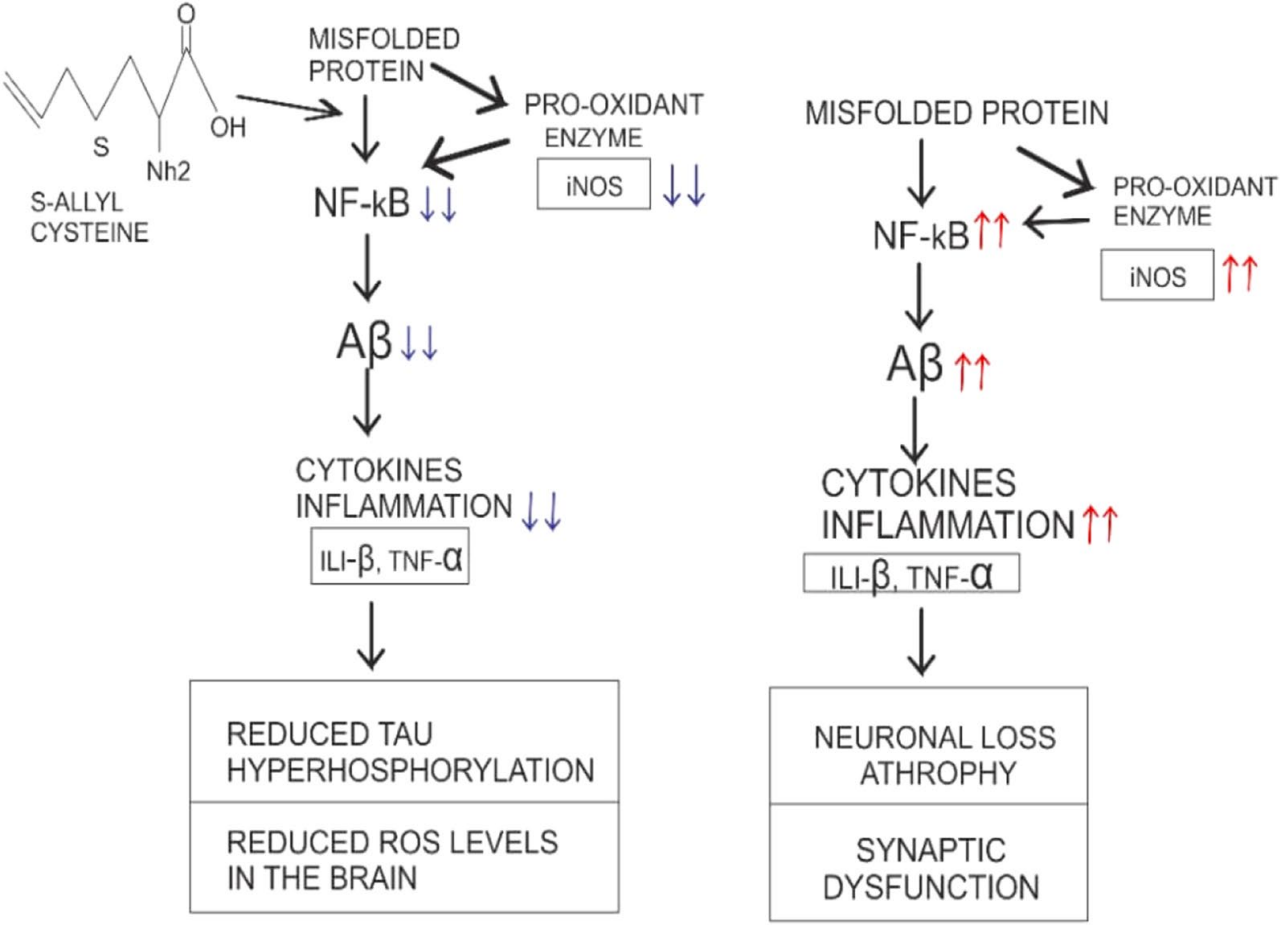
Describing the role of S-allyl cysteine on misfolded protein (a characteristic of tauopathies) ↓↓: downregulation ↑↑: upregulation

More acknowledgeable, is the unique flavor attributed to freshly prepared garlic, which is notably due to the presence of alkyl thiosulfinates, thiosulfinates, s-alkyl-substituted cysteine sulfoxide derivatives, pyruvate, and NH3 [154]. Several studies have highlighted the therapeutic potential attributed to garlic; thus, the medicinal efficacy of garlic is well renowned. These include immunodialatory, antitumor, anti-inflammatory, antioxidant, antimicrobial, cardioprotective, anticancer and neuroprotective potential [155–158]. As earlier explained, neurodegenerative tauopathies occurs when misfolded proteins upregulate the inflammatory cytokines, activating the NF-Kβ signaling cascade and further downregulate ERK, CREB and Akt pathways. However, research has affirmed the efficacy of garlic via its bioactive compound s-allyl cysteine (SAC). SAC was found to inhibit TNFα and IL1β [159], downregulates NF-Kβ signaling cascades [160, 161] and further limits the activity of iNOS [162]. Furthermore, the neuroprotective potential of Allium sativum has been explored. Several such studies include remediation of neuronal damage by SAC [163, 164], neuroprotective potential in mice model [165], improved behavioral activity in mice [166] and finally memory enhancing effects of aged garlic extract [167]. While Aβ induced neurotoxicity remains an indicator of neurodegenerative tauopathies, Jeong et al. [168] further stressed the ameliorative potential of garlic. Finally, Allium sativum is classified as a member of GRAS (Generally Recognized as Safe) by FDA, due to its limited adverse effect, as such it is acknowledgeable to encourage the use of garlic for human consumption (Fig. 2).

### Withania somnifera

**Scheme 2:**
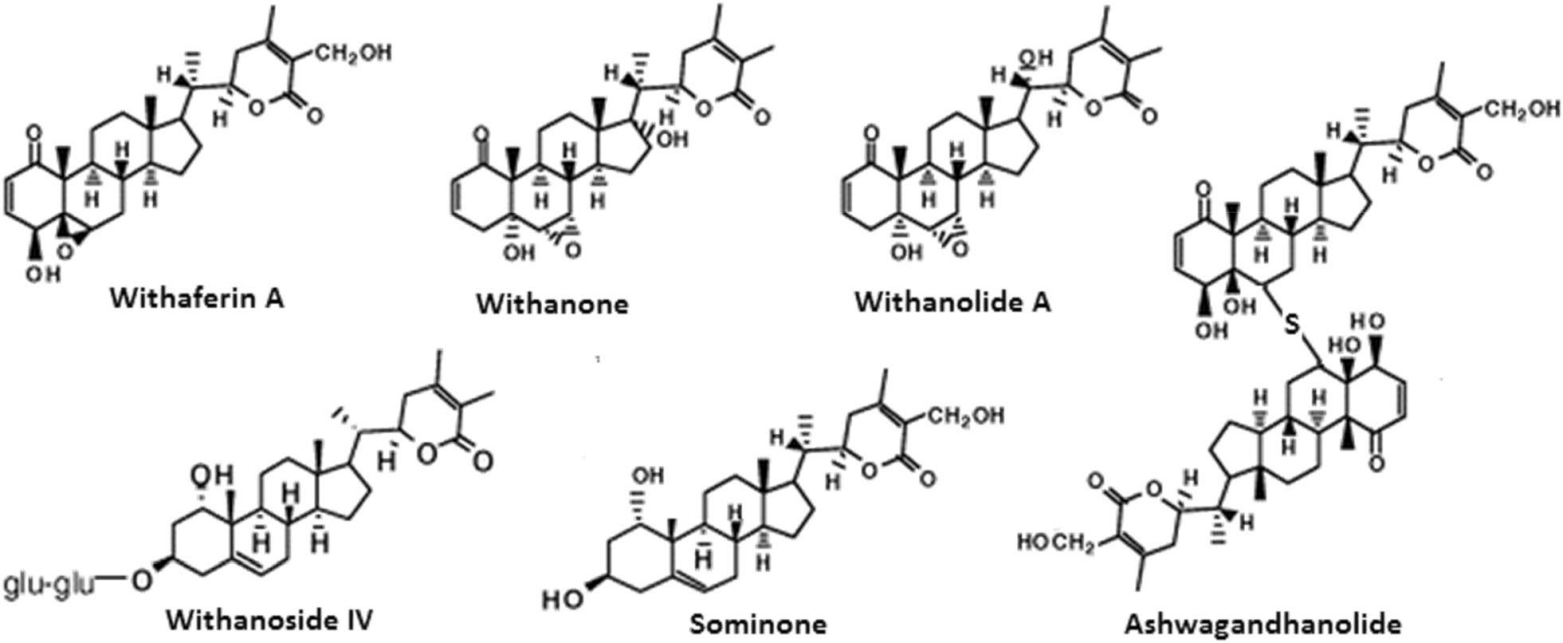
Bioactive compounds in *Withania somnifera*

Withania somnifera (WS) is popularly referred to as Ashwagandha, belonging to the family Solanaceae and identified by a small green shrub with long roots. Distributed across south Africa, middle east India and China, WS have had its part exploited since ages in the management of various human diseases [169, 170]. Biochemically composed of a combined steroidal alkaloids and lactones called Withanolide [171]. Withanolide are side chain steroidal nucleus decorated by six membered lactone rings [172, 173], consisting of essential alkaloids such as withananine, tropine, choline, anaferine amidst others. Evaluation of the toxicological properties of WS by several studies and FDA, considers WS as a safe drug for human consumption, having been explored and revealed antioxidant capabilities, sedative, anti-inflammatory potential, memory enhancing capabilities, pain relief and antimicrobial potential [174–176]. The neuroprotective potential of WS has undergone extensive studies (Scheme 2).

Some of which includes; enhancement of dendrites formation in neuroblastoma cell [177], memory enhancement via withanoside IV induced RET modulation [178], regeneration and reconstruction of axons and synapses in mice damaged brain [179] and protection against cellular brain damage [180]. Recall that neurodegenerative tauopathies are characterized by the onset of Aβ toxicity, however, WS bioactive compound withanolide protected against pheochromocytoma cells against Aβ toxicity and inhibited fibril formation [181, 182].

**Fig. 3:**
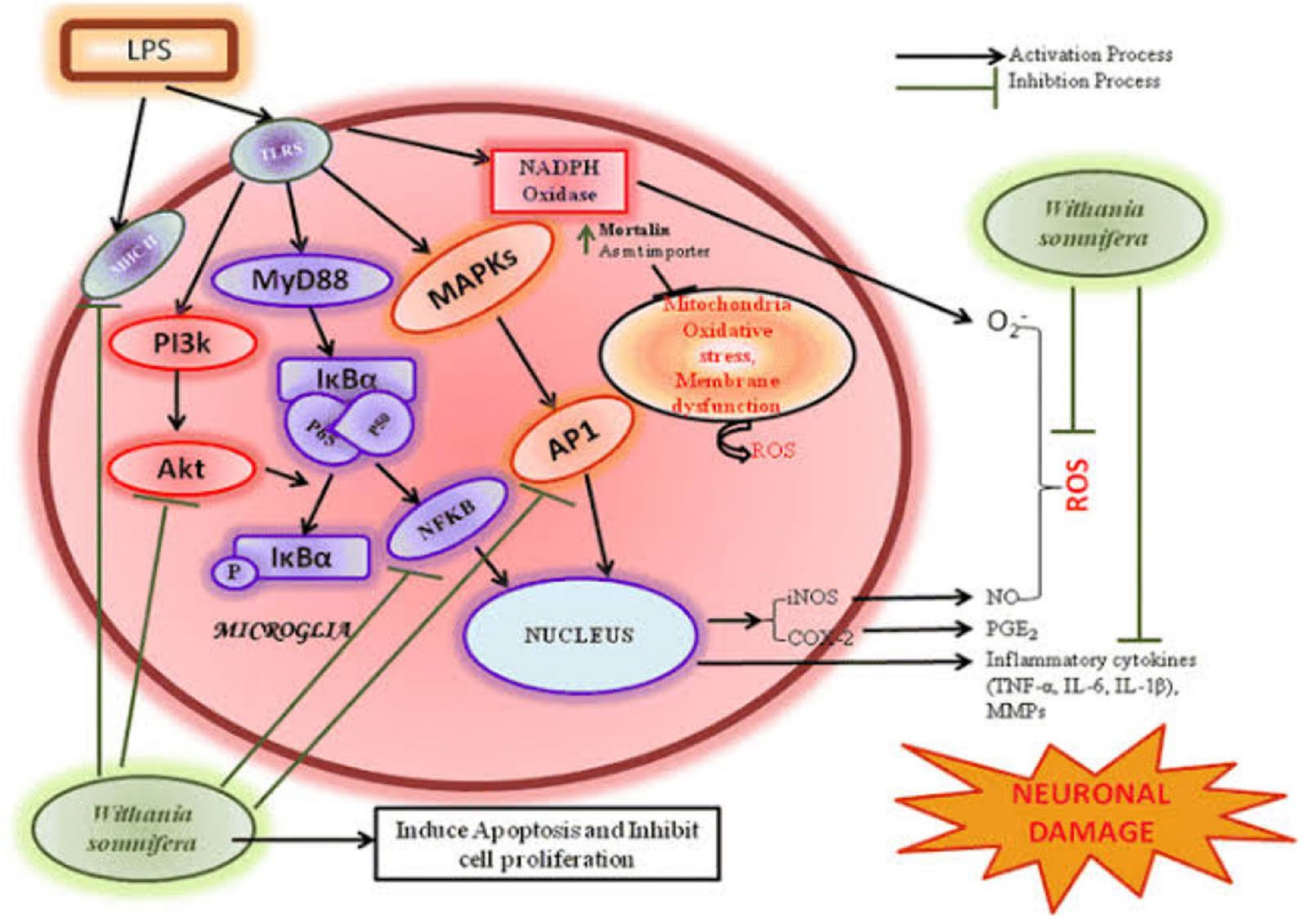
Illustrating the possible role of Withania somnifera as an anti-neuroinflammatory nutraceutical [186]

Furthermore, the antidementia potential of WS in mice model of AD shows upregulation of low-density lipoprotein receptor in AD pathology reversal and improved cognitive functioning [183], all pointing at the neuroprotective potential of WS in the amelioration of neurodegenerative tauopathies. Finally, WS has been affirmed in regulating oxidative stress, inhibiting lipid peroxidation [184], and increasing ROS scavenging activity by upregulating SOD and catalase activity [185] (Fig. 3).

### Bacopa monnieri

**Scheme 3:**
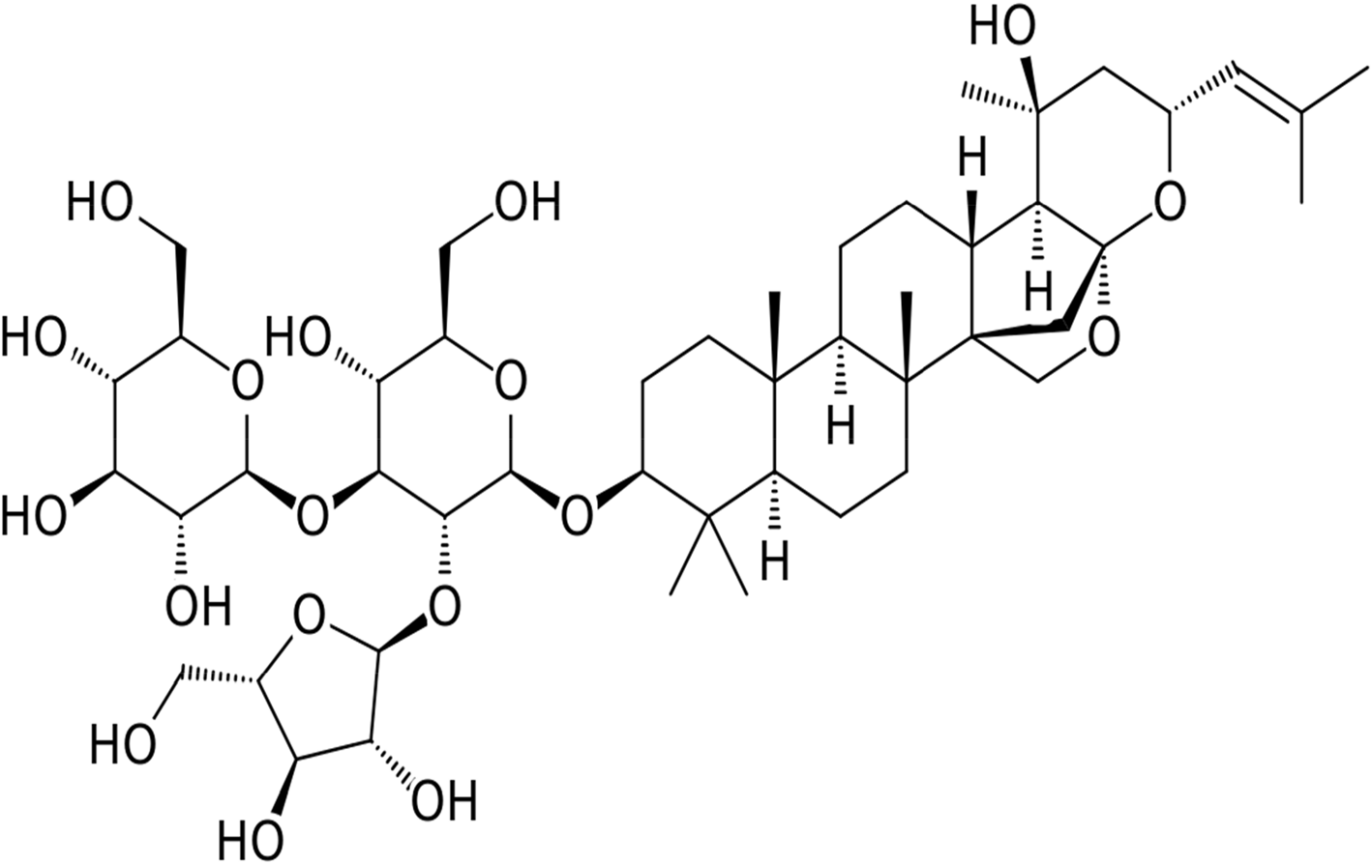
Structure of Bacoside

Bacopa monnieri (Brahimi; BM) is a perennial, highly branched, succulent herb characterized by a fleshy, spatulate and sessile leaves, singly arranged pale blue flower, bivalve ovoid capsule fruits and minute seeds [187]. They are distributed across India, Africa, Pakistan and China; mostly grown for medicinal purposes [188]. Acclaiming a height of about 2–3 feet tall, Brahimi has been phytochemically analyzed to be a multi-functional compound with multiple bioactive compounds [189]. Some of which includes triterpenoids saponins, glycosides, alkaloids and alcohols. Looking inward, their alkaloid contains brahmine, nicotine and herpestine, their glycoside contains pseudojujugbogeun (3-O-[α-1-arabinofuranosyl (1–2) β-d-glucopyranosyl]), and their triterpenoids contains Bacoside A3 (chemically known as triterpenoid saponin 3-β-[O-β-d-glucopyranosyl (1–3)-O-[α-l-arabinofuranosy (1–2)]-O-β-d-glucopyranosyl)oxy]), and Bacoside A [190] (Scheme 3).

However, Bacoside A and Bacoside B is attributed to be responsible for the neuroprotective potential of Brahimi, with respect to the presence of several saponins including Bacoside A3, bacopaside, jujubogenin and bacoposaponin C [191]. Notable therapeutic efficacy of BM includes neuroprotection against AD [192], memory dysfunction [193] and dementia [194]. However, further studies have explained its role in enhancing cognitive functioning [195], anti-inflammation capabilities [196], hepatoprotective potential [197] and anti-aging capabilities [198]. A hallmark of oxidative stress induced cellular damage is the oxidation of intracellular proteins resulting in neurodegenerative diseases [199], however, BM reduces protein carbonyl levels in cytosol and mitochondrial fragments in prevention of oxidative damage [200]. More so, the irreversible damage caused of cellular organelles caused by lipid peroxidation has showed BM as an efficient cellular protective compound in protecting the prefrontal cortex, striatum in rat models [201] and reducing MDA levels [202]. Furthermore, the maintenance of SOD levels [203, 204], glutathione activity [200, 205] and glutathione peroxidase activity reduction [205–207]. Designated as a memory booster, and considered as a nootropic herbal drug, BM has showed its efficacy in igniting cognitive functioning [192, 208]. While the decline in in cognitive functioning still remains as a manifestation in neurodegenerative tauopathies, the improvement in logical thinking, sense of judgement and problem-solving skills relays the therapeutic potential of BM. Similarly, the aggregation of Aβ and tau proteins in AD and other tauopathies have been shown to be on decline in WS and BM treatment [209]. This efficacy is attributed to the neuroprotective potential of Bacoside A, against Aβ induced cytotoxicity in SH-S454 cells [210]. Terneehooheep et al. [211], in his study reflected on the ameliorating potential of BM extract on tauopathies suggests that BM prevents the hyperphosphorylation of tau proteins and as such attenuates tau-mediated toxicity. Neuroinflammation of tauopathies is triggered by the deposition of tau protein aggregates which in principle activates proinflammatory cytokines TNF α and IL1β; i.e. the exposure of Aβ fibers, triggers the activation of proinflammatory cytokines, causing neuroinflammation [212]. However, a study by Viji et al. [213], explained the anti-inflammatory potential of BM via the inhibition of NF-Kβ and ERK signaling cascades, thus improving learning, memory and consequently synapse functioning. More so, the inhibition of TNF α by the bioactive compound triterpenoids and bacoside has further been elucidated [214]. Finally, the efficacy of BM in the reduction of 1L1β in an in vivo brain damage rat study, results in improved cognitive functioning [215].

### Role of tumeric in neurodegenrative tauopathies

**Scheme 4:**
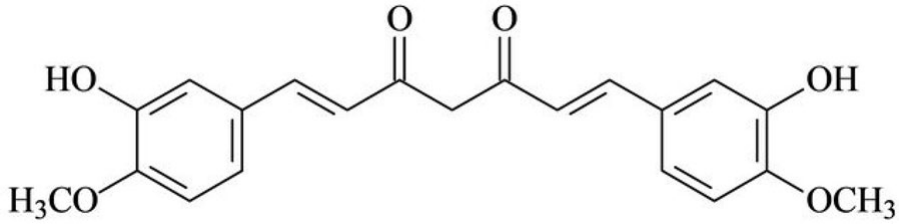
Structure of curcumin

Turmeric, also known as *Curcuma longa* is a plant prominently grown in Southeast Asia, including China. The dried rhizomes are eaten as spice and is related to the family of ginger. Curcumin is an essential component of turmeric, responsible for its brownish-yellow color. Amongst its other bioactive compounds are desmethoxycurcumin and bisdemethoxycurcumin which can collectively be known as curcuminoids [216]. Curcumin, with the chemical nomenclature ((1E,6E)-1,7-Bis(4-hydroxy-3-methoxyphenyl)-1,6-heptadiene-3,5-dione) is classified as a beta-diketone molecule enabling both chemical and medicinal activities such as anti- oxidant, anti- inflammatory and anti- cancer activities, as well as a "cleanser of the body"[217] (Scheme 4).

Curcumin has been of great interest because of its strong efficacy and affinity for fibrillar amyloid proteins [218]. Anti-amyloid properties of curcumin result in decreasing Aβ production, inhibiting Aβ aggregation and promotion of Aβ clearance. Curcumin binds readily with other β pleated proteins such as A-Synuclein, p-tau & AD and prion proteins [219, 220]. The major chemical feature of Aβ is the presence of two aromatic end group which has effect on its activity if altered. Due to the lipophilic property of curcumin, brain tissue enables it to bypass the blood brain barrier and binds to plaques thus, inhibiting the aggregation of Aβ proteins [221].

One of the major characteristics of tauopathies is inflammation of nerve cells. Inflammation is therefore reduced by curcumin mainly by inhibiting Egr-1 DNA binding activity in THP-1 cells (a major inflammatory transcription factor) [222, 223]. Curcumin also serve as an anti- inflammatory agent by inhibiting the enzyme cyclooxygenase (Cox-2),5- Lipoxygenase(5-Lox), enzymes responsible for biosynthesis of prostaglandins [224]. NF-kB, a neuroinflammatory marker protein is also downregulated by Turmeric [225], as well as the expression of IL-1, IL-6 and TNF-α in LPS-stimulated BV2 microglia, is reduced by curcumin [226, 227]*.* Aβ aggregate and generation of ROS in various neurodegenerative diseases can be produced by heavy metal such as copper (cu), zinc (zn), lead(pb) and manganese [228, 229]. Considering the chemical structure of curcumin, the presence of two phenolic group and one active methylene group makes it a perfect binding agent for any metal ion attached by coordinate bonds preventing neurotoxicity [230]. A coordinate bonds is formed when curcumin binds with copper, iron and zinc causing the non-availability of metals to produce amyloid protein aggregation.

There is also increase in the expression of NF-KB levels by heavy metals causing neuroinflammation. Since it has been noted that curcumin check inflammation by obstructing NF-kB levels, possibly, this is carried out by metal chelation [231, 232].

**Fig. 4:**
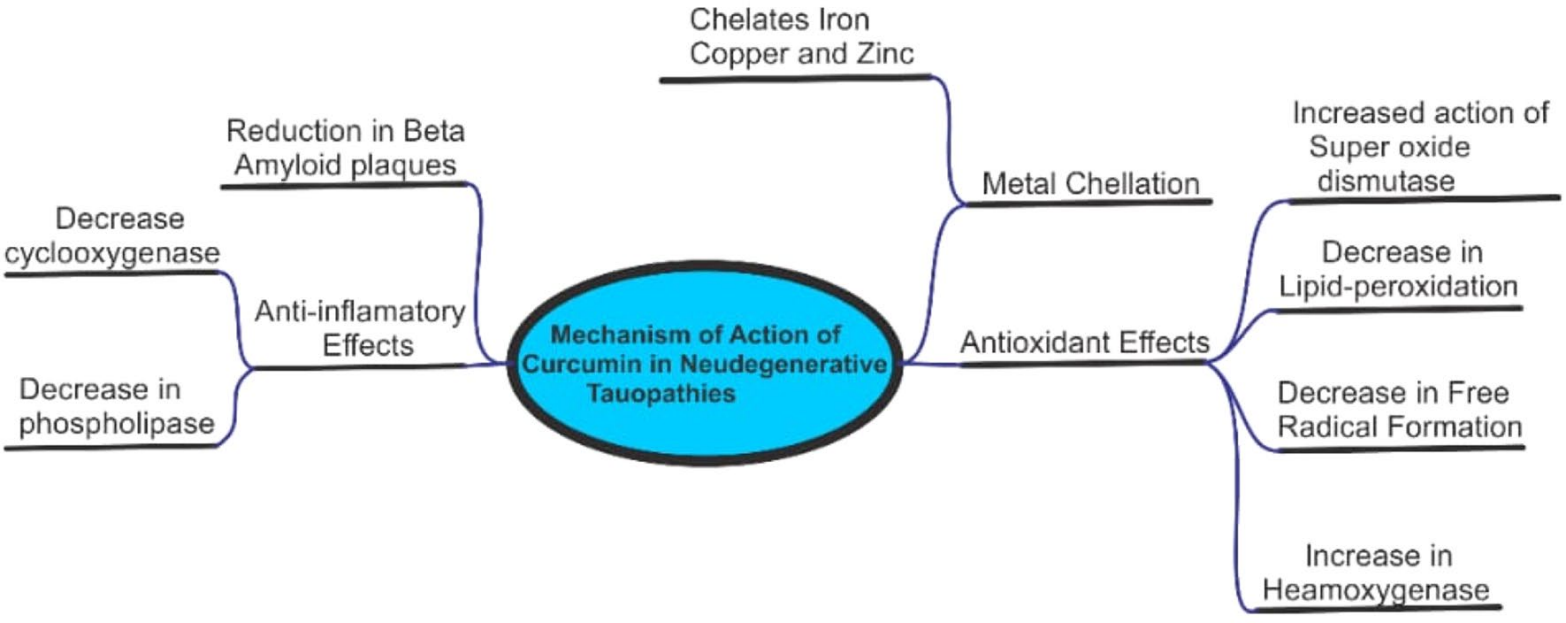
Schematic representation of the mechanism of curcumin action in neurodegenerative tauopathies

A study carried out by Kozmon and Tvaroska 2015, revealed the association between Aβ peptide and copper ions and curcumin and was observed that curcumin not only chelated heavy metals (cu, pb, zn) but also form "curcumin—cu2 +—Aβ and curcumin-Aβ complexes when curcumin is directly attached to Aβ thereby decreasing toxic B-sheets structure [233]*.* Turmeric shows properties of antioxidants i.e. curcumin, the major active component protects cells from damage caused by free radicals. This damage occurs when there is accumulation of ROS which in return affect polyunsaturated fatty acids. Antioxidant properties of curcumin help to increase superoxide dismutase, glutathione peroxidase, glutathione transferase activities which in return preserves the level of glutathione and decreases malonaldehyde accumulation [234]. Along with the antioxidant effects, curcumin has also been noted to eliminate NO-based radicals [235]. An In-vivo study revealed suppression in the level of carbonyl protein in transgenic mouse models exhibiting human Alzheimer's disease gene when administered curcumin. Thus, it was deduced that curcumin prevent oxidative damage caused by lipid peroxidation, which induce carbonyl compounds (hydroxynomenal), while also inhibiting the activity of AP1; a transcription factor involved in expression of amyloid [236] (Fig. 4).

### Role of ginseng in neurodegenerative tauopathies

**Scheme 5:**
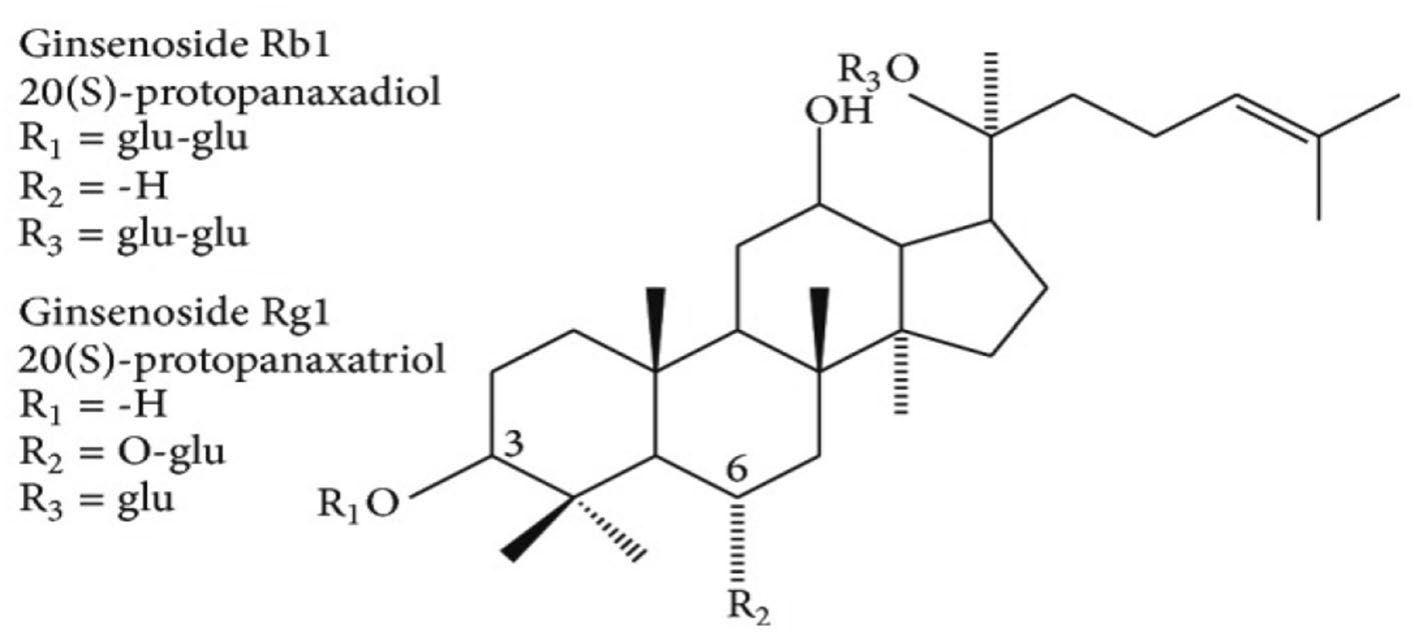
Structure of ginsenosides

Ginseng is known to be one of the popular traditional plants of the family are Araliaceae (perennial plant) and genus "panax". It has its name originated from "Jen Sheng" a Chinese word meaning "man herb" due to the shape of the root which is human like shape [237], “ panax” a Greek word meaning "all heal", this shows that it can cure all kind of diseases [238]. The commonly studied Ginseng are "Panax ginseng", "Panax quinquefolium" and Panax notoginseng [239] exhibiting a lot of biological effects [237]. The active constituents of ginseng called "ginsenosides" shows neuroprotective effects and enhances memory [240] (Scheme 5).

Aβ is coined for peptides constituting 36–43 amino acid residues. Aβ formation is from Aβ precursor protein (APP) present in neurons through successive hydrolysis of protein with an enzyme beta secretase 1(BACE1) and γ-secretase [241].

**Fig. 5:**
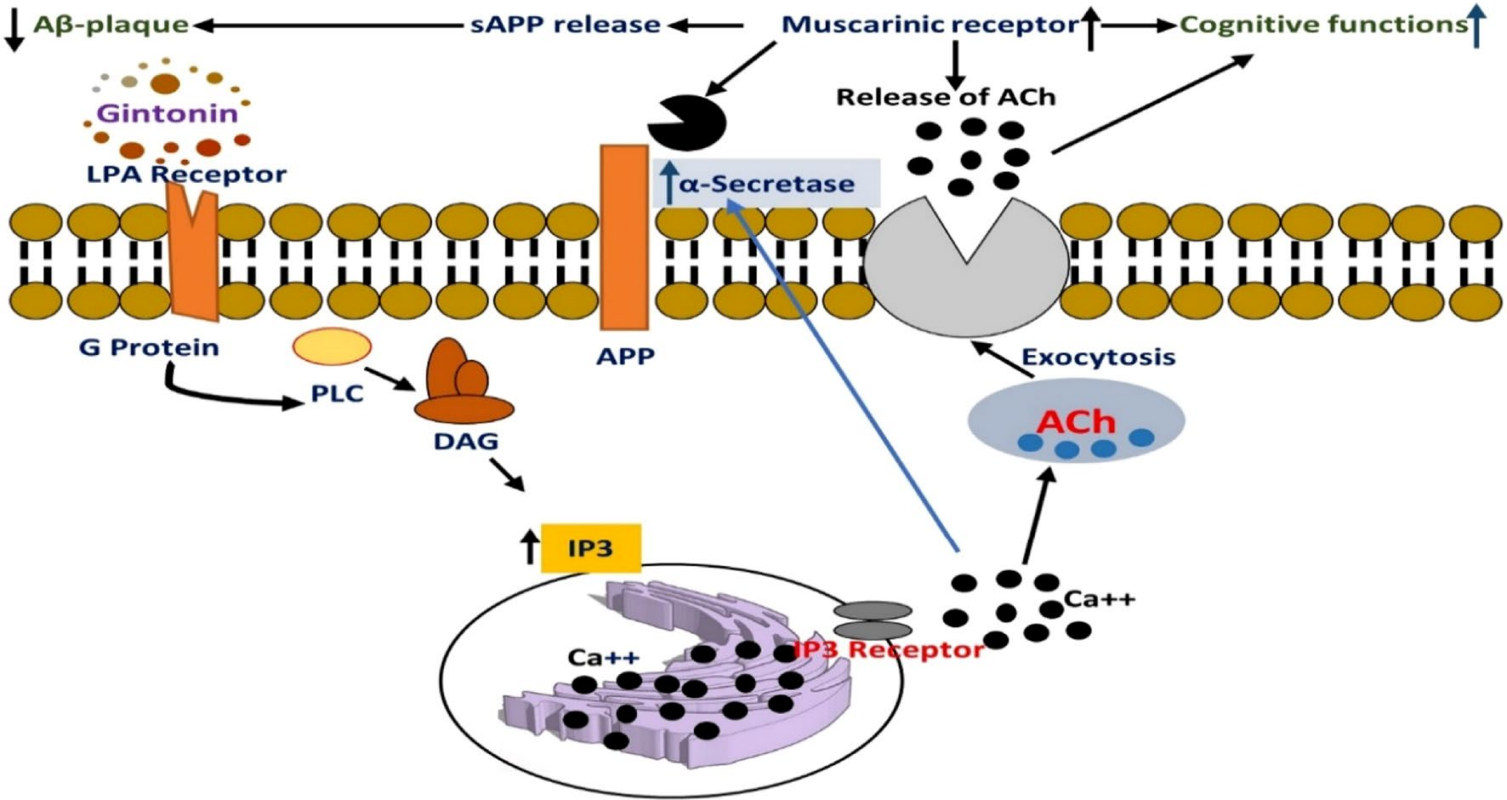
Graphical illustration of gintonin effects against tauopathies. Gintonin stimulates the release of acetylcholine, thus upregulating chat expression and stimulating cognitive functioning of neural cells (source: [247])

Some ginsenosides are BACE1 inhibitors. Their inhibiting ability is in decreasing order: Rc > Rg1 > Rg2 > Rb1 > Rg3 > Re [241]. Gintonin acts in ameliorating tauopathies via the reduction of accumulated amyloid plaque. Hyperphosphorylated tau protein inhibits Panax ginseng by upregulating the activities of phosphatase activities in SY5Y cells. However, Ginsenosides Rd and Rb1 reduces hyperphosphorylated tau by increasing phosphatase 2A level (PP2A) [242]. Rg1 also reduces Aβ formation and decreases hyperphosphorylated tau. One of the major treatments of AD is a principal Acetylcholine (ACh) neurotransmitters in which cognition and memory process is reduced in AD [237]. This neurotransmitter is terminated by an enzyme known as Acetylcholinesterase (AChE), and butyryl cholinesterase (BChE), which is present in patients with AD as well as inhibiting of choline acetyltransferase (ChAT) activity (an enzyme responsible for ACh metabolism) [243].Studies have shown that Rb1,Rb2, Rc,Re,Rg1 and Rg3 have a reducing property on AChE and BChE [243, 244]. Rg2 also decreases intracellular Ca2 + level and ROS which is caused by the presence of Aβ also reduce lipid peroxidation that is produced by glutamate. Ginsenosides Rb1 and Rb5(in-vitro) and genocide Rd(in-vivo) are capable of reducing the expression of anti-inflammatory factors such as IL-1B, IL-6, TNF-α by inhibiting the activation of NF-kβ [245]. Cox-2 and NOS 2, a major enzyme in the biosynthesis of prostaglandins and neurotransmitter that helps in learning and memory mechanism respectively is seen to be increased in models of tauopathies and the level of NOS1 reduces. Nonetheless, management of tauopathies using ginsenosides Rg5 reduces the level of Cox-2 and NOS 2 [245], while the increase in the level of NOS 1 increases and Cox-2 decreases with the treatment of Rb1 [246] (Fig. 5).

### Role of physical activity in neurodegenerative tauopathies

Physical activity and exercise is defined as contraction of skeletal muscle expressing the movement of the body, yielding energy expenditure. Nevertheless, the major difference between physical activity and exercise is that the former includes various types of movement while the latter is a type of physical activities which helps in the improvement and maintenance of physical fitness by planned and structural activities [248]. Due to the pleiotropic favorable impact on human tissues e.g. Muscles, vascular, heart and brain, constant physical activities are considered as an important component of a healthy living resulting in inhibition and resistance of various chronic pathological disease such as cardiovascular, metabolic and neurodegenerative diseases [249]. Some benefits of exercise have been found to ameliorate special learning, working memory, executive and cognitive function [250, 251]. In addition, it causes many neurobiological operations e.g. regulating giving off of neurotransmitters [12], regulating hormones and neurotrophic factor levels [252] to be produced on brain areas activating both acute and chronic biological effects. Many factors affecting growth required in the growth of correct brain tissue, e.g. Fibroblast growth factor-2 (FGF-2) [253], insulin –like growth factor-1 (IGF-1) [254], vascular endothelial growth factor (VEGF)[255], and brain derived neurotrophic factor (BDNF)[256] are through exercise increased in level. The decrease of OS at brain level in addition to the regulation of these factors [257], and the regulation of cell death and inauguration of neurogenesis in precise area of the brain [258], is accountable for defensive consequences of exercise as regards neurodegenerative diseases [12]. Much likely defensive procedure, essential for the impact of physical activity on dementia danger have been suggested as well as rise in brain derived neurotrophic factor (BDNF), decrease in cardiovascular disease and metabolic syndrome risk, with rise in flow of cerebral blood [259]. Eminently, there is an association of decrease in danger of a number of dementia types, in addition to AD emerging to be most sensitive with regulation of the afore-mentioned elements via increase in physical activity [260]. Consequently, it's rational to postulate that variations of mechanism and deterioration of Aβ and tau are significantly essential procedures expected amid exercise and AD risk. Extracellular amyloid plaques are formed by the aggregation of Aβ peptides resulting to decrease in cognitive functions and neuronal death in tauopathies. Major components of amyloid plaques is the lengthy, and more fibrillar isoform of Aβ, Aβ 1–42[261]. It had been identified by autopsy evaluation of AD brains that there is amyloid deposit initially detected in the cingulate cortex, accompanied by the temporal and parietal cortices and the caudate. There is formation of plagues located in occipital, sensory and motor brain region at the subsequent stage of this neuropathological process [262]. Identifying individuals with AD, it has been proven within living human studies that quantification of Aβ in the cerebrospinal fluid and brain (through positron emission tomography, PET, and Aβ binding agent) have proven responsive and precised [263]. Instead of complete inhibition, there is likelihood that exercise hold back and also decrease Aβ accumulation. Which reflects the importance of exercise being commenced before start of symptoms (preclinical period). Additionally, Um et al. [264] for seventeen months, measured Aβ, noticing impact of exercise on decreased Aβ 1–42 deposition. Following physical activity and exercise, it has been reported from many animal studies that there is reduction in phosphorylation of tau present in brain and tau pathology [265–269], and reduction in hippocampal tau pathology [266, 267, 270] using intervention varying from 2 to 5 months and 2–9 months have been observed from studies. In the effect of physical activity and exercise on brain tau, it is uncertain whether specific aspects of intervention involve in a key function from the findings made. In respect of tau reduction, as it may be postulated that greater potency running can evoke better favorable impact. Yet, research applying forced running [266, 267] and voluntary wheel running probably at a reduced intensity [265, 270] detect result on both phosphorylation and accumulation. Over a certain period of 2 months, it is feasible that steady aerobic exercise evokes decrease in tau using animal specimen. However, a review disclosed rise in insoluble tau levels and phosphorylation of tau at the C terminus implementing physical activities [271]. Notwithstanding, there is a complex correlation amid exercise, inflammation and neurodegenerative processes: Decreased AD pathology is linked to increase and decrease in inflammatory marker, having ranging (increase to decrease) adaptive inflammatory response to exercise [272, 273]. In detecting brain tau, varying techniques were used, such as western blotting [267, 268, 271, 274], enzyme linked immunosorbent assays [270], Sarkozy extraction [265], and immunofluorescence [269]. Although it still remains obscure, how these varying procedures could impact the reported results because of the all-round procedures used. According to Gratuze et al. (2017) [265], described none impact of voluntary wheel moving on some tau kinases (GSK3, CDK5, C-JUN N terminal kinases (JNK) and calmodulin -dependent protein kinase 11 (camk11)) nor phosphatase (which dephosphorylate tau in vitro) in their animals. It has been shown that a lack in 2 cholesterol binding proteins, Niemann-pick disease, type C1 (NPC1) and type 2 (NPC2), give rise to tau Pathology. Belarbi and partners in their mice following voluntary wheel running, observed upregulation of NPC1 and NPC2mRNA [270]. There is a further requirement of extensive research considering the numerous potentially mediating factors such as kinases and phosphatases. Baker et al. [275] discovered a lower CSF tau in healthy group only to be associated with high-intensity physical activity: It is certain that insufficient intensity or volume of exercise might be undertaken by MCI group to discover a connection with CSF tau. The relationship between habitual physical activity levels and CSF biomarkers of AD has been evaluated with the use of objectively measured physical activity (actigraphy),[276]; a lesser proportion of total tau/Aβ1-42 and phosphorylated tau /Aβ 1–42 (showing a less cerebral pathology) was seen in people spending adequate time participating in moderate physical activity. Lately, Brown et al. [277] reported the greatest levels of exercise showing lesser extent of PET calculated tau in reasonably elderly persons and also, pathological extent of tau protein is not yet reached even with those having "greater" extent of tau in the brain. With the recent studies on tau neuroimaging, the association between physical activity and brain tau in people is a great research work in the coming years (Table 2).

**Table 2:** Illustrating the types of exercise, impacts and effects on tau activity

Exercise type	Duration	Physical activities	Impacts	Safety tips	Changes to tau activity	Refernces
Endurance excerise	Minimum of 150 min per week	Jogging, dancing, swimming, biking, hills climbing, playing tennis	Improves fitness levels Aids daily tasks Improves the health of the heart, lungs and circulatory system Prevent cardiovascular diseases	Easy walking before and after endurance exercise Prevent injuries Drink fluids that makes you sweat	Inhibition of free radical activities in the brain Activates AKT/PKB cascades, thus consequently reduces tau hyperphosphorylation	[278, 279]
Strength exercise	Minimum of two days per week	Push-up, squats, digging, weight lifting, use of resistance bands	Increases bone density Improves cardiac function Keeps muscle strong Prevents falls and fall related injuries	Never hold breath during strength exercise Breathe out as you lift and breathe in as you relax Prevent injuries	Downregulation of phospho-PKA levels and upregulating PKC levels Reduces tau hyperphosphorylation	[280]
Flexibility exercise	2 to 3 days per week at 15- 30 s repeated 2–4 times	Shoulder rolls, neck & shoulder release, chest mobiliser, ankle stretch, calf stretch	Improves flexibility Improves balance Stretches muscles	Warm up prior to stretching Don’t stretch if it hurts Breathe normally while holding a stretch	Downregulation of phosphor-ERK1/2, phosphor JNK and p38 levels were thus inhibiting the over expression of tau protein	[280]
Balance exercise	2 to 3 days per week	Step up, Heel to toe walk, one-leg stand, sideways walking	Prevent falls Improves core muscles Improves balance	Use a sturdy chair when necessary Prevent injuries	Inhibition of GSK3β, a kinase mediating tau hyperphosphorylation	[281]

## Conclusion

As explored in this study, tau have proved essential in triggering neurodegenerative diseases cascade, representing a great hurdle to human health of the twenty-first century. Since induction of oxidative stress, instability of microtubule, upregulation of proinflammatory cytokines and cognitive repression are involved in the progression of neurodegenerative tauopathies, potential ameliorating strategies to traditional pharmacological treatments (such as dietary administration of nutraceuticals and moderate physical activity) should focus on modulation of microtubule tau activity. Essentially, nutraceutical-containing nano systems, for targeted neuronal activity have a great potential neuronal remediation strategy, as they could bypass blood brain barriers for targeting neuronal cells, thus enhancing the bioactive effects. Tailored interventions with targeted nutraceuticals to reduce neuroinflammation, improve cognitive functioning and to induce enzymes with a great antioxidant potential, together with the activation of exercise in increasing the levels of growth factors involved in correct brain tissue development, such as fibroblast growth factor-2 (FGF- 2), insulin-like growth factor-1 (IGF-1), vascular endothelial growth factor (VEGF), and brain derived neurotrophic factor (BDNF),thus contributing to the improvement of the pathological profile of diverse oxidation-related brain neuropathology. However, despite the promising relationship between tau, physical activity, nutraceuticals and neurodegenerative tauopathies, the challenge to conform in vivo study model to human model still remains and is still to be fully elucidated.

### Future perspective

Apart from explored therapeutic target, biomarkers and drugs available, the quest for complete treatment of tauopathies still persist. While the synergism between pharmacological and non-pharmacological treatments is applaudable, the role of secondary metabolites in Chinese nutraceuticals is praiseworthy. However more robust preclinical and clinical trials against novel drug target with minimal adverse effect for the complete suppression of tauopathies modulation should be carried out.

Transformation achievements belonging to tauopathies such as therapeutic employment of neuro-imaging, identification of molecular cascades involving mediating tau-induced neuronal loss, atrophy, development of tau-based immunotherapy and antisense oligonucleotides are rapidly evolving the biological comprehension, management and diagnosis in treatment of tauopathies, thus offering a great hope for the future.

Finally, nanotechnology has made great stride in diseases managements and drug design in recent years. Thus, future studies should explore the role of nanotechnology in neurodegenerative tauopathies as well as creating a novel therapeutic target.

## Data Availability

Not applicable.

## Abbreviations

MDA5: Melanoma differentiation-associated protein 5
NF-κB: Nuclear factor kappa light chain enhancer of activated B cells
IRF: Interferon regulatory transcription factor
IFNAR: Interferon α and β receptor 1
JAK: Janus Kinase
Aβ: β- Amyloid peptide
AD: Alzheimer’s disease
FGF-2: Fibroblast growth factor-2
IGF-1: Insulin-like growth factor-1
VEGF: Vascular endothelial growth factor
NFTs: Neurofibrillary Tangles
HD: Huntington’s disease
PHFs: Paired helical filaments
BDNF: Brain derived neurotrophic factor
NPC1: Niemann-Pick disease, type C1
NPC2: Niemann-Pick disease type C2
CamKII: Calmodulin-dependent protein kinase II
JNK: C-Jun N terminal kinases
PET: Positron emission tomography
BDNF: Brain-derived neurotrophic factor
FGF-2: Fibroblast growth factor-2
IGF-1: Insulin-like growth factor-1
MAPT: Microtubule associated protein tau
VEGF: Vascular endothelial growth factor
FTDP-17: Frontotemporal dementia with Parkinsonism-17
TNFα: Tumor necrosis factor alpha
IL1β: Interleukin 1 beta
BM: Bacopa monnieri
WS: Withania somniferous
SAC: S-allyl cysteine
GRAS: Generally Recognized as Safe
GSK3β: Glycogen synthase kinase 3 beta
CREB: CAMP response element-binding protein
Akt: Protein Kinase A/ Protein Kinase B: PKA/PKB
MAPK/ERK: Mitogen-activated protein kinase
SAPK/JNK: Stress-activated protein kinase
AgD: Argyrophilic grain disease
ArG: Argyrophilic grain
MOBP: Myelin associated oligodendrocyte basic proteins
MS: Multiple sclerosis
CBS: Corticobasal syndrome
CBD: Corticobasal degeneration
SAPK/JNK: Stress activated protein kinase
PSP: Progressive supranuclear palsy
OS: Oxidative stress
PSP-CBD: PSP with corticobasal degeneration
FTLD: Frontotemporal lobar degeneration
CBD: Corticobasal degeneration
PiD: Pick's disease
AGD: Argyrophilic grain disease
FDA: Food and Drug Administration
GDP: Gross Domestic Product
CHO: Carbohydrate
EGR1: Early Growth Response 1
THP-1: Human leukemia monocytic cell line
DAG: Diacylglycerol
IP3: Inositol trisphosphate
LPA receptor: Lysophosphatidic acid receptors
PLC: Phospholipase C

## References

[1] World Health Organization, Dementia, 2017. https://www.who.int/news-room/factsheets/detail/dementia.

[2] Rodolfo S, Grossardt BR, Bower JH, Ahlskog JE, Rocca WA (2013). Incidence and pathology of synucleinopathies and tauopathies related to parkinsonism. JAMA Neurol..

[3] Alavi-Naini SM, Soussi-Yanicostas N (2015). Tau hyperphosphorylation and oxidative stress, a critical vicious circle in neurodegenerative tauopathies?. Oxid Med Cell Long.

[4] Weingarten MD, Lockwood AH, Hwo S-Y, Kirschner MW (1975). A protein factor essential for microtubule assembly. Proc Natl Acad Sci.

[5] Rodríguez-Martín T, Cuchillo-Ibáñez I, Noble W, Nyenya F, Anderton BH, Hanger DP (2013). Tau phosphorylation affects its axonal transport and degradation. Neurobiol Aging.

[6] Wang Y, Mandelkow E (2016). Tau in physiology and pathology. Nat Rev Neurosci.

[7] Orr ME, Sullivan AC, Frost B (2017). A brief overview of tauopathy: causes, consequences, and therapeutic strategies. Trends Pharmacol Sci.

[8] Mietelska-Porowska A, Wasik U, Goras M, Filipek A, Niewiadomska G (2014). Tau protein modifications and interactions: their role in function and dysfunction. Int J Mol Sci.

[9] Hutton M, Lendon CL, Rizzu P, Baker M, Froelich S, Houlden H, Pickering-Brown S, Chakraverty S, Isaacs A, Grover A (1998). Association of missense and 5′-splice-site mutations in tau with the inherited dementia FTDP-17. Nature.

[10] Selkoe DJ (2002). Alzheimer’s disease is a synaptic failure. Science.

[11] Cummings DM, Liu W, Portelius E, Bayram S, Yasvoina M, Ho SH, Smits H, Ali SS, Steinberg R, Pegasiou CM, James OT (2015). First effects of rising amyloid-β in transgenic mouse brain: synaptic transmission and gene expression. Brain..

[12] Hamilton GF, Rhodes JS (2015). Exercise regulation of cognitive function and neuroplasticity in the healthy and diseased brain. Prog Mol Biol Transl Sci.

[13] Meeusen R, De Meirleir K (1995). Exercise and brain neurotransmission. Sports Med.

[14] Ding Q, Vaynman S, Akhavan M, Ying Z, Gomez-Pinilla F (2006). Insulin-like growth factor I interfaces with brain-derived neurotrophic factor-mediated synaptic plasticity to modulate aspects of exercise-induced cognitive function. Neuroscience.

[15] Uysal N, Kiray M, Sisman AR, Camsari UM, Gencoglu C, Baykara B, Cetinkaya C, Aksu I (2015). Effects of voluntary and involuntary exercise on cognitive functions, and VEGF and BDNF levels in adolescent rats. Biotech Histochem.

[16] Vaynman S, Gomez-Pinilla F (2005). License to run: exercise impacts functional plasticity in the intact and injured central nervous system by using neurotrophins. Neurorehabil Neural Repair.

[17] Kim DH, Ko IG, Kim BK, Kim TW, Kim SE, Shin MS, Kim CJ, Kim H, Kim KM, Baek SS (2010). Treadmill exercise inhibits traumatic brain injury-induced hippocampal apoptosis. Physiol Behav.

[18] Koren S, Galvis-Escobar S, Abisambra JF (2020). Tau-mediated dysregulation of RNA: Evidence for a common molecular mechanism of toxicity in frontotemporal dementia and other tauopathies. Neurobiol Dis.

[19] Goedert M, Spillantini MG, Jakes R, Rutherford D, Crowther RA (1989). Multiple isoforms of human microtubule-associated protein tau: sequences and localization in neurofibrillary tangles of Alzheimer’s disease. Neuron.

[20] Bartel DP (2009). MicroRNAs: target recognition and regulatory functions. Cell.

[21] Bryan JB, Nagle BW, Doenges KH (1975). Inhibition of tubulin assembly by RNA and other polyanions: evidence for a required protein. Proc Natl Acad Sci USA..

[22] Cruz A, Mamta V, Benjamin W (2019). The pathophysiology of tau and stress granules in disease. Adv Exp Med Biol..

[23] Johnson ECB, Dammer EB, Duong DM (2018). Deep proteomic network analysis of Alzheimer's disease brain reveals alterations in RNA binding proteins and RNA splicing associated with disease. Mol Neurodegener.

[24] Seyfried NT, Gozal YM, Donovan LE, Herskowitz JH, Dammer EB, Xia Q, Ku L, Chang J, Duong DM, Rees HD, Cooper DS (2016). Changes in the detergent-insoluble brain proteome linked to amyloid and tau in Alzheimer's Disease progression. Proteomics..

[25] Grundke-Iqbal I, Iqbal K, Tung YC, Quinlan M, Wisniewski HM, Binder LI (1986). Abnormal phosphorylation of the microtubule-associated protein tau (tau) in Alzheimer cytoskeletal pathology. Proc Natl Acad Sci USA..

[26] Apicco DJ, Zhang C, Maziuk B, Jiang L, Ballance HI, Boudeau S, Ung C, Li H, Wolozin B (2019). Dysregulation of RNA splicing in tauopathies. Cell Rep..

[27] Hsieh YC, Guo C, Yalamanchili HK, Abreha M, Al-Ouran R, Li Y, Dammer EB, Lah JJ, Levey AI, Bennett DA, De Jager PL (2019). Tau-Mediated Disruption of the Spliceosome Triggers Cryptic RNA Splicing and Neurodegeneration in Alzheimer's Disease. Cell Rep..

[28] Maziuk BF, Apicco DJ, Cruz AL (2018). RNA binding proteins co-localize with small tau inclusions in tauopathy. Acta Neuropathol Commun.

[29] Wolozin B, Ivanov P (2019). Stress granules and neurodegeneration. Nat Rev Neurosci.

[30] Piao YS, Hayashi S, Wakabayashi K, Kakita A, Aida I, Yamada M, Takahashi H (2002). Cerebellar cortical tau pathology in progressive supranuclear palsy and corticobasal degeneration. Acta Neuropathol.

[31] Evans HT, Benetatos J, van Roijen M, Bodea LG, Götz J. Decreased synthesis of ribosomal proteins in tauopathy revealed by non-canonical amino acid labelling. EMBO J. 2019;38(13): 101174. doi*: *10.15252/embj.2018101174*.* (**Epub 2019 May 22**). 10.15252/embj.2018101174 PMC6600635 31268600

[32] Kobayashi S, Tanaka T, Soeda Y, Almeida OF, Takashima A (2017). Local Somatodendritic Translation and Hyperphosphorylation of Tau Protein Triggered by AMPA and NMDA Receptor Stimulation. EBioMedicine..

[33] Li C, Gotz J (2017). Somatodendritic accumulation of Tau in Alzheimer's disease is promoted by Fyn-mediated local protein translation. EMBO J..

[34] Marotta CA, Majocha RE, Coughlin JF, Manz HJ, Davies P, Ventosa-Michelman M, Chou WG, Zain SB, Sajdel-Sulkowska EM (1986). Transcriptional and translational regulatory mechanisms during normal aging of the mammalian brain and in Alzheimer's disease. Prog Brain Res..

[35] Ohno M (2014). Roles of eIF2alpha kinases in the pathogenesis of Alzheimer's disease. Front Mol Neurosci..

[36] Santacruz K, Lewis J, Spires T, Paulson J, Kotilinek L, Ingelsson M, Guimaraes A, DeTure M, Ramsden M, McGowan E (2005). Tau suppression in a neurodegenerative mouse model improves memory function. Science.

[37] Sydow A, Van der Jeugd A, Zheng F, Ahmed T, Balschun D, Petrova O, Drexler D, Zhou L, Rune G, Mandelkow E (2011). Tau-induced defects in synaptic plasticity, learning, and memory are reversible in transgenic mice after switching off the toxic Tau mutant. J Neurosci.

[38] Patterson KR, Remmers C, Fu Y, Brooker S, Kanaan NM, Vana L, Ward S, Reyes JF, Philibert K, Glucksman MJ (2011). Characterization of prefibrillar Tau oligomers in vitro and in Alzheimer disease. J Biol Chem.

[39] Gerson JE, Sengupta U, Lasagna-Reeves CA, Guerrero- Munoz MJ, Troncoso J, Kayed R (2014). Characterization of tau oligomeric seeds in progressive supranuclear palsy. Acta Neuropathol Commun.

[40] Berger Z, Roder H, Hanna A, Carlson A, Rangachari V, Yue M, Wszolek Z, Ashe K, Knight J, Dickson D (2007). Accumulation of pathological tau species and memory loss in a conditional model of tauopathy. J Neurosci.

[41] Yoshiyama Y, Higuchi M, Zhang B, Huang SM, Iwata N, Saido TC, Maeda J, Suhara T, Trojanowski JQ, Lee VM (2007). Synapse loss and microglial activation precede tangles in a P301S tauopathy mouse model. Neuron.

[42] Roy DS, Arons A, Mitchell TI, Pignatelli M, Ryan TJ, Tonegawa S (2016). Memory retrieval by activating engram cells in mouse models of early Alzheimer’s disease. Nature.

[43] Sohn PD, Tracy TE, Son HI, Zhou Y, Leite RE, Miller BL, Seeley WW, Grinberg LT, Gan L (2016). Acetylated tau destabilizes the cytoskeleton in the axon initial segment and is mislocalized to the somatodendritic compartment. Mol Neurodegener.

[44] Morris M, Knudsen GM, Maeda S, Trinidad JC, Ioanoviciu A, Burlingame AL, Mucke L (2015). Tau post-translational modifications in wild-type and human amyloid precursor protein transgenic mice. Nat Neurosci.

[45] Przedborski S, Vila M, Jackson-Lewis V (2013). Series Introduction: Neurodegeneration: what is it and where are we?. J Clin Investig.

[46] Lee VM, Goedert M, Trojanowski JQ (2001). Neurodegenerative tauopathies. Annu Rev Neurosci.

[47] Götz J, Halliday G, Nisbet RM (2019). Molecular Pathogenesis of the Tauopathies.

[48] Goedert M, Ghetti B, Spillantini MG (2012). Frontotemporal dementia: implications for understanding Alzheimer disease. Cold Spring Harbor Perspectives in Medicine.

[49] Höglinger GU, Respondek G (2018). Kovacs, G, New classification of tauopathies. Revue Neurologique.

[50] Kovacs GG (2015). Invited review: Neuropathology of tauopathies: principles and practice. Neuropathol Appl Neurobiol.

[51] Rösler TW, Marvian AT, Brendel M, Nykänen NP, Höllerhage M, Schwarz SC, Hopfner F, Koeglsperger T, Respondek G, Schweyer K, Levin J. Four-repeat tauopathies. Progr Neurobiol. 2019. doi: 10.1016/j.pneurobio.2019.101644*.* [Epub ahead of print] 10.1016/j.pneurobio.2019.101644 31238088

[52] Selkoe DJ, Hardy J (2016). The amyloid hypothesis of Alzheimer's disease at 25 years. EMBO Mol Med.

[53] Yamada T, McGeer P, McGeer E (1992). Appearance of paired nucleated, Tau-positive glia in patients with progressive supranuclear palsy brain tissue. Neurosci Lett.

[54] Dickson DW, Kouri N, Murray ME, Josephs KA (2011). Neuropathology of Frontotemporal Lobar Degeneration-Tau (FTLD-Tau). J Mol Neurosci.

[55] E. Visidi, T. Dam, M. Juneja, L. Li, H. Krzywy, S. Eaton, S. Chen, S. Hall, A. Dilley. Prevalence and characteristics of patients with progressive supranuclear palsy (PSP) in US health insurance claims data (abstract). Mov Disord. 2018; 33 (suppl2). http://www.mdsabstracts.org/abstract/prevalence-and-characteristics-of-patients-with-progressive-supranuclear-palsy-psp-in-us-health-insurance-claims-data/. Accessed 14 Sept 2020.

[56] Takigawa H, Ikeuchi T, Aiba I, Morita M, Onodera O, Shimohata T, Tokuda T, Murayama S, Nakashima K (2016). Japanese Longitudinal Biomarker Study in PSP and CBD (JALPAC): a prospective multicenter PSP/CBD cohort study in Japan. Parkinsonism Relat Disord.

[57] Litvan I, Agid Y, Calne D, Campbell G, Dubois B, Duvoisin RC, Goetz CG, Golbe LI, Grafman J, Growdon JH, Hallett M, Jankovic J, Quinn NP, Tolosa E, Zee DS (1996). Clinical research criteria for the diagnosis of progressive supranuclear palsy (Steele-Richardson-Olszewski syndrome): report of the NINDS-SPSP international workshop. Neurology.

[58] Respondek G, Stamelou M, Kurz C, Ferguson LW, Rajput A, Chiu WZ, van Swieten JC, Troakes C, Al Sarraj S, Gelpi E, Gaig C (2014). The phenotypic spectrum of progressive supranuclear palsy: a retrospective multicenter study of 100 definite cases. Mov Disord..

[59] Respondek G, Roeber S, Kretzschmar H, Troakes C, Al-Sarraj S, Gelpi E, Gaig C, Chiu WZ, van Swieten JC, Oertel WH, Höglinger GU (2013). Accuracy of the National Institute for Neurological Disorders and Stroke/Society for Progressive Supranuclear Palsy and neuroprotection and natural history in Parkinson plus syndromes criteria for the diagnosis of progressive supranuclear palsy. Mov Disord.

[60] Josephs KA, Katsuse O, Beccano-Kelly DA, Lin W-L, Uitti RJ, Fujino Y, Boeve BF, Hutton ML, Baker MC, Dickson DW (2006). Atypical progressive supranuclear palsy with corticospinal tract degeneration. J Neuropathol Exp Neurol.

[61] Nagao S, Yokota O, Nanba R, Takata H, Haraguchi T, Ishizu H, Ikeda C, Takeda N, Oshima E, Sakane K, Terada S (2012). Progressive supranuclear palsy presenting as primary lateral sclerosis but lacking parkinsonism, gaze palsy, aphasia, or dementia. J Neurol Sci..

[62] Ling H, O’Sullivan SS, Holton JL, Revesz T, Massey LA, Williams DR, Paviour DC, Lees AJ (2010). does corticobasal degeneration exist?. A clinicopathological re-evaluation, Brain.

[63] Compta Y, Valldeoriola F, Tolosa E, Rey MJ, Marti MJ, Valls-Sole J (2007). long lasting pure freezing of gait preceding progressive supranuclear palsy: a clinicopathological study. Mov Disord.

[64] Williams DR, Holton JL, Strand K, Revesz T, Lees AJ (2007). Pure akinesia with gait freezing: a third clinical phenotype of progressive supranuclear palsy. Mov Disord.

[65] Kanazawa M, Tada M, Onodera O, Takahashi H, Nishizawa M, Shimohata T (2013). Early clinical features of patients with progressive supranuclear palsy with predominant cerebellar ataxia. Parkinsonism Relat Disord.

[66] Koga S, Josephs KA, Ogaki K, Labbe C, Uitti RJ, Graff-Radford N, Van Gerpen JA, Cheshire WP, Aoki N, Rademakers R (2016). Cerebellar ataxia in progressive supranuclear palsy: An autopsy study of PSP-C. Mov Disord.

[67] Litvan I, Lees PS, Cunningham CR, Rai SN, Cambon AC, Standaert DG, Marras C, Juncos J, Riley D, Reich S, Hall D, Kluger B, Bordelon Y, Shprecher DR (2016). Environmental and occupational risk factors for progressive supranuclear palsy: Case-control study. Mov Disord.

[68] Caparros-Lefebvre D, Golbe LI, Deramecourt V, Maurage CA, Huin V, Buee-Scherrer V, Obriot H, Sablonniere B, Caparros F, Buee L, Lees AJ (2015). A geographical cluster of progressive supranuclear palsy in northern France. Neurology.

[69] Caparros-Lefebvre D, Sergeant N, Lees A, Camuzat A, Daniel S, Lannuzel A, Brice A, Tolosa E, Delacourte A, Duyckaerts C (2002). Guadeloupean parkinsonism: a cluster of progressive supranuclear palsy-like tauopathy. Brain.

[70] Lannuzel A, Ruberg M, Michel PP (2008). Atypical parkinsonism in the Caribbean island of Guadeloupe: etiological role of the mitochondrial complex I inhibitor annonacin. Mov Disord.

[71] Kouri N, Ross OA, Dombroski B, Younkin CS, Serie DJ, Soto-Ortolaza A, Baker M, Finch NCA, Yoon H, Kim J (2015). Genome-wide association study of corticobasal degeneration identifies risk variants shared with progressive supranuclear palsy. Nat Commun.

[72] Rohrer JD, Paviour D, Vandrovcova J, Hodges J, De Silva R, Rossor MN (2011). Novel L284R MAPT mutation in a family with an autosomal dominant progressive supranuclear palsy syndrome. Neurodegen Dis.

[73] Ogaki K, Li Y, Takanashi M, Ishikawa K-I, Kobayashi T, Nonaka T, Hasegawa M, Kishi M, Yoshino H, Funayama M (2013). Analyses of the MAPT, PGRN, and C9orf72 mutations in Japanese patients with FTLD, PSP, and CBS. Parkinsonism Relat Disord.

[74] Fernandez-Botran R, Ahmed Z, Crespo FA, Gatenbee C, Gonzalez J, Dickson DW, Litvan I (2011). Cytokine expression and microglial activation in progressive supranuclear palsy. Parkinsonism Relat Disord.

[75] Cantuti-Castelvetri I, Keller-McGandy CE, Albers DS, Beal MF, Vonsattel J-P, Standaert DG, Augood SJ (2002). Expression and activity of antioxidants in the brain in progressive supranuclear palsy. Brain Res.

[76] Sian J, Dexter DT, Lees AJ, Daniel S, Agid Y, Javoy-Agid F, Jenner P, Marsden CD (1994). Alterations in glutathione levels in Parkinson's disease and other neurodegenerative disorders affecting basal ganglia. Ann Neurol.

[77] Ferrer I, Blanco R, Carmona M, Puig B (2001). Phosphorylated mitogen-activated protein kinase (MAPK/ERK-P), protein kinase of 38 kDa (p38-P), stress-activated protein kinase (SAPK/JNK-P), and calcium/calmodulin-dependent kinase II (CaM kinase II) are differentially expressed in tau deposits in neurons and glial cells in tauopathies. J Neural Transm.

[78] Tolosa E, Litvan I, Hoglinger GU, Burn D, Lees A, Andres MV, Gomez-Carrillo B, Leon T, Del Ser T (2014). A phase 2 trial of the GSK-3 inhibitor tideglusib in progressive supranuclear palsy. Mov Disord.

[79] Steele JC, Richardson JC, Olszewski J (1964). Progressive supranuclear palsy. Arch Neurol.

[80] Rebeiz JJ (1968). Kolodny EH Corticodentatonigral degeneration with neuronal achromasia: a progressive disorder of late adult life. Arch Neurol.

[81] Winter Y (2010). Incidence of Parkinson’s disease and atypical parkinsonism: Russian population-based study. Mov Disord.

[82] Bergeron C, Davis A, Lang AE (1998). Corticobasal ganglionic degeneration and progressive supranuclear palsy presenting with cognitive decline. Brain Pathol.

[83] Watts, R. L., Mirra, S. S. & Richarson, E. P. Jr in *Movement Disorders III: Blue Books of Practical Neurology* (eds Marsden, C. D. & Fahn, S.) Butterworth–Heinemann, Oxford,.1994:13:282–299.

[84] Riley DE, Lang AE (1988). Corticobasal ganglionic degeneration (CBGD): further observations in six additional cases. Neurology.

[85] Ouchi H, Toyoshima Y, Tada M (2013). Pathology and sensitivity of current clinical criteria in corticobasal syndrome. Mov Disord.

[86] Ling H, Kovacs GG, Vonsattel JPG (2016). Astrogliopathy predominates the earliest stage of corticobasal degeneration pathology. Brain.

[87] Armstrong MJ, Litvan I, Lang AE (2013). Criteria for the diagnosis of corticobasal degeneration. Neurology.

[88] Kempuraj D, Thangavel R, Selvakumar GP (2017). Brain and peripheral atypical inflammatory mediators potentiate neuroinflammation and neurodegeneration. Front Cell Neurosci.

[89] Murray ME, Kouri N, Lin W-L (2014). Clinicopathologic assessment and imaging of tauopathies in neurodegenerative dementias. Alzheimers Res Ther.

[90] Ferrer I, López-González I, Carmona M (2014). Glial and neuronal tau pathology in tauopathies: characterization of disease specific phenotypes and tau pathology progression. J Neuropathol Exp Neurol.

[91] Ayers JI, Giasson BI, Borchelt DR (2017). Prion-like spreading in tauopathies. Biol Psychiatry.

[92] Querol-Vilaseca M, Colom-Cadena M, Pegueroles J (2017). YKL-40 (chitinase 3-like I) is expressed in a subset of astrocytes in Alzheimer’s disease and other tauopathies. J Neuroinflamm.

[93] Lee SE, Rabinovici GD, Mayo MC (2011). Clinicopathological correlations in corticobasal degeneration. Ann Neurol.

[94] Kouri N, Ross OA, Dombroski B (2015). Genome-wide association study of corticobasal degeneration identifies risk variants shared with progressive supranuclear palsy. Nat Commun.

[95] Bukki J, Nubling G, Lorenzl S (2016). Managing advanced progressive supranuclear palsy and corticobasal degeneration in a palliative care unit: admission triggers and outcomes. Am J Hosp Palliat Med.

[96] Lamb R, Rohrer JD, Lees AJ, Morris HR (2016). Progressive supranuclear palsy and corticobasal degeneration: pathophysiology and treatment options. Curr Treat Options Neurol.

[97] Cho JW, Lee JH (2014). Suppression of myoclonus in corticobasal degeneration by levetiracetam. J Mov Disord.

[98] Eschlböck S, Krismer F, Wenning GK (2016). Interventional trials in atypical parkinsonism. Parkinsonism Relat Disord.

[99] Gallyas F (1971). Silver staining of Alzheimer’s neurofibrillary changes by means of physical development. Acta Morphol Acad Sci Hung.

[100] Ishihara K, Araki S, Ihori N (2005). Argyrophilic grain disease presenting with frontotemporal dementia: a neuropsychological and pathological study of an autopsied case with presenile onset. Neuropathology.

[101] Maurage CA, Sergeant N, Schraen-Maschke S (2003). Diffuse form of argyrophilic grain disease: a new variant of four-repeat tauopathy different from limbic argyrophilic grain disease. Acta Neuropathol.

[102] Braak H, Braak E (1987). Argyrophilic grains: characteristic pathology of cerebral cortex in cases of adult-onset dementia without Alzheimer changes. Neurosci Lett.

[103] Braak H, Braak E (1998). Argyrophilic grain disease: frequency of occurrence in different age categories and neuropathological diagnostic criteria. J Neural Transm.

[104] Schultz C, Koppers D, Sassin I (1998). Cytoskeletal alterations in the human tuberal hypothalamus related to argyrophilic grain disease. Acta Neuropathol.

[105] Knopman DS, Parisi JE, Salviati A (2003). Neuropathology of cognitively normal elderly. J Neuropathol Exp Neurol.

[106] Tolnay M, Clavaguera F (2004). Argyrophilic grain disease: a late-onset dementia with distinctive features among tauopathies. Neuropathology.

[107] Tolnay M, Sergeant N, Ghestem A (2002). Argyrophilic grain disease and Alzheimer’s disease are distinguished by their different distribution of tau protein isoforms. Acta Neuropathol.

[108] Ferrer I, Barrachina M, Tolnay M (2003). Phosphorylated protein kinases associated with neuronal and glial tau deposits in argyrophilic grain disease. Brain Pathol.

[109] Morris M, Maeda S, Vossel K, Mucke L (2011). The many faces of tau. Neuron.

[110] Frost B, Götz J, Feany MB (2015). Connecting the dots between taudysfunction and neurodegeneration. Trends Cell Biol..

[111] Bakota L, Brandt R (2016). Tau biology and tau-directed therapies for Alzheimer’sdisease. Drugs.

[112] Hyman BT, Phelps CH, Beach TG, Bigio EH, Cairns NJ, Carrillo MC (2012). National Institute on Aging-Alzheimer's Association guidelines for the neuropathologic assessment of Alzheimer's disease. Alzheimer's Dementia.

[113] Jack CR, Bennett DA, Blennow K, Carrillo MC, Dunn B, Haeberlein SB (2018). NIA-AA research framework: toward a biological definition of Alzheimer's disease. Alzheimers Dement.

[114] Sancesario GM, Bernardini S (2015). how many biomarkers to discriminate neurodegenerative dementia?. Crit Rev Clin Lab Sci.

[115] Molinuevo JL, Ayton S, Batrla R, Bednar M, Bittner T, Cummings J (2018). Current state of Alzheimer's fluid biomarkers. Acta Neuropathol.

[116] Marsh SE, Blurton-Jones M. Examining the mechanisms that link beta-amyloid and alpha-synuclein pathologies. Alzheimers Res Ther 2012; 4(2):11. doi: 10.1186/alzrt109 (eCollection 2012). 10.1186/alzrt109 PMC4054672 22546279

[117] Savica R, Beach TG, Hentz JG, Sabbagh MN, Serrano GE, Sue LI (2019). Lewy body pathology in Alzheimer's disease: a clinicopathological prospective study. Acta Neurol Scand.

[118] McAleese KE, Walker L, Erskine D, Thomas AJ, McKeith IG, Attems J (2017). TDP-43 pathology in Alzheimer's disease, dementia with Lewy bodies and ageing. Brain Pathol.

[119] Chang XL, Tan MS, Tan L, Yu JT (2016). The role of TDP-43 in Alzheimer's disease. Mol Neurobiol.

[120] Duka V, Lee J-H, Credle J, Wills J, Oaks A, Smolinsky C, Shah K, Mash DC, et al. Identification of the sites of tau hyperphosphorylation and activation of tau kinases in synucleinopathies and Alzheimer’s diseases. PLoS ONE. 2013;8(9): 75025. doi: 10.1371/journal.pone.0075025. (**eCollection 2013**). 10.1371/journal.pone.0075025 PMC3779212 24073234

[121] Reynolds CH, Betts JC, Blackstock WP, Nebreda AR, Anderton BH (2000). Phosphorylation sites on tau identified by nano electrospray mass spectrometry: differences in vitro between the mitogen-activated protein kinases ERK2, c-Jun N-terminal kinase and P38, and glycogen synthase kinase-3β. J Neurochem.

[122] Wang W, Qiao Y, Li Z (2018). New insights into modes of GPCR activation. Trends Pharmacol Sci.

[123] Angulo E, Casadó V, Mallol J, Canela EI, Viñals F, Ferrer I, Lluis C, Franco R (2003). A1 adenosine receptors accumulate in neurodegenerative structures in Alzheimer's disease and mediate both amyloid precursor protein processing and tau phosphorylation and translocation. Brain Pathol.

[124] Xia M, Hyman BT (2002). GROα/KC, a chemokine receptor CXCR2 ligand, can be a potent trigger for neuronal ERK1/2 and PI-3 kinase pathways and for tau hyperphosphorylation—a role in Alzheimer's disease?. J Neuroimmunol.

[125] Mishra S, Palanivelu K (2008). The effect of curcumin (turmeric) on Alzheimer’s disease: an overview. Ann Indian Acad Neurol.

[126] Liu Z, Zhang A, Sun H (2017). Two decades of new drug discovery and development for Alzheimer’s disease. RSC Adv.

[127] Scarpini E, Scheltens P, Feldman H (2003). Treatment of Alzheimer’s disease: current status and new perspectives. Lancet Neurol.

[128] Graham WV, Bonito-Oliva A, Sakmar TP (2017). Update on Alzheimer’s disease therapy and prevention strategies. Annu Rev Med.

[129] Mirra SS, Schneider JA, Gearing M, Watts RL, Koller WC (1997). Neuropathology of movement disorders: an overview. Movement disorders: neurologic principles and practice.

[130] Ruberg M, Javoy-Agid F, Hirsch E (1985). Dopaminergic and cholinergic lesions in progressive supranuclear palsy. Ann Neurol.

[131] Robbins TW, James M, Owen AM (1994). Cognitive deficits in progressive supranuclear palsy, Parkinson’s disease, and multiple system atrophy in tests sensitive to frontal lobe dysfunction. J Neurol Neurosurg Psychiatry.

[132] Särkämö T, Tervaniemi M, Laitinen S, Forsblom A, Soinila S, Mikkonen M, Autti T, Silvennoinen HM, Erkkilä J, Laine M, Peretz I, Hietanen M (2008). Music listening enhances cognitive recovery and mood after middle cerebral artery stroke. Brain.

[133] Ikeda K, Akiyama H, Arai T, Matsushita M, Tsuchiya K, Miyazaki H (2000). Clinical aspects of argyrophilic grain disease. Clin Neuropathol.

[134] Tolnay M, Monsch AU, Probst A (2001). Argyrophilic grain disease. A frequent dementing disorder in aged patients. Adv Exp Med Biol.

[135] Lockwood B. Nutraceuticals. 2nd ed London _ Grayslake (USA): Pharmaceutical Press; 2007; 71(5): 99.

[136] "Supplement Makers Touting Cures for Alzheimer's and Other Diseases Get F.D.A. Warning". The New York Times. 11 February 2019. Retrieved 2019–05–11.

[137] "Labeling & Nutrition". The Food and Drug Administration, US Department of Health and Human Services. 5 October 2016. Retrieved 2016–10–11.

[138] CRN Consumer Survey on dietary supplements, 2015. http://www.crnusa.org/CRNconsumersurvey/2015/CRN-ConsumerSurvey-onepager.pdf.

[139] Schwab S, Heier M, Schneider A (2014). The use of dietary supplements among older persons in southern Germany results from the KORA-age study. J Nutr Health Aging.

[140] del Balzo V, Vitiello V, Germani A (2014). A cross sectional survey on dietary supplements consumption among Italian tee-agers. PLoS ONE.

[141] Barnes K, Ball L, Desbrow B (2016). Consumption and reasons for use of dietary supplements in an Australian university population. Nutrition..

[142] Calderon-Garciduenas L, Franco-Lira M, Mora-Tiscareno A, Medina-Cortina H, Torres-Jardon R, Kavanaugh M (2013). Early Alzheimer’s and Parkinson’s disease pathology in urban children: friend versus foe responses–it is time to face the evidence. Biomed Res Int.

[143] Cheng B, Gong H, Xiao H, Petersen RB, Zheng L, Huang K (2013). Inhibiting toxic aggregation of amyloidogenic proteins: a therapeutic strategy for protein misfolding diseases. Biochim Biophys Acta.

[144] Masters SL, O’Neill LA (2011). Disease-associated amyloid and misfolded protein aggregates activate the inflammasome. Trends Mol Med.

[145] Nakamura T, Cho DH, Lipton SA (2012). Redox regulation of protein misfolding, mitochondrial dysfunction, synaptic damage, and cell death in neurodegenerative diseases. Exp Neurol.

[146] Iqbal K, Liu F, Gong CX, Alonso Adel C, Grundke-Iqbal I (2009). Mechanisms of tau-induced neurodegeneration. Acta Neuropathol..

[147] Matsuzaki K, Kato K, Yanagisawa K (2010). Abeta polymerization through interaction with membrane gangliosides. Biochim Biophys Acta.

[148] Hernandez CM, Dineley KT (2012). Alpha7 nicotinic acetylcholine receptors in Alzheimer’s disease: neuroprotective, neurotrophic or both?. Curr Drug Targets.

[149] Sultan MT, Butt MS, Qayyum MM, Suleria HA (2014). Immunity: plants as effective mediators. Crit Rev Food Sci Nutr.

[150] Butt MS, Sultan MT, Butt MS, Iqbal J (2009). Garlic: nature’s protection against physiological threats. Crit Rev Food Sci Nutr.

[151] Singh BB, Vinjamury SP, Der-Martirosian C, Kubik E, Mishra LC, Shepard NP (2007). Ayurvedic and collateral herbal treatments for hyperlipidemia: a systematic review of randomized controlled trials and quasi-experimental designs. Altern Ther Health Med.

[152] Tapiero H, Townsend DM, Tew KD (2004). Organosulfur compounds from alliaceae in the prevention of human pathologies. Biomed Pharmacother.

[153] Macpherson LJ, Geierstanger BH, Viswanath V, Bandell M, Eid SR, Hwang S (2005). The pungency of garlic: activation of TRPA1 and TRPV1 in response to allicin. Curr Biol.

[154] Krest I, Glodek J, Keusgen M (2000). Cysteine sulfoxides and alliinase activity of some *Allium* species. J Agric Food Chem.

[155] Borlinghaus J, Albrecht F, Gruhlke MCH, Nwachukwu ID, Slusarenko AJ (2014). Allicin: Chemistry and biological properties. Molecules.

[156] Kumar R, Chhatwal S, Arora S, Sharma S, Singh J, Singh N, Khurana A (2013). Antihyperglycemic, antihyperlipidemic, anti-inflammatory and adenosine deaminase-lowering effects of garlic in patients with type 2 diabetes mellitus with obesity. Diabetes Metab Syndr Obes..

[157] Lanzotti V, Scala F, Bonanomi G (2014). Compounds from Allium species with cytotoxic and antimicrobial activity. Phytochem Rev.

[158] Zhu JW, Chen T, Guan J, Liu WB, Liu J (2012). Neuroprotective effects of allicin on spinal cord ischemia-reperfusion injury via improvement of mitochondrial function in rabbits. Neurochem Int.

[159] da Lee Y, Li H, Lim HJ, Lee HJ, Jeon R, Ryu JH (2012). Anti-inflammatory activity of sulfur-containing compounds from garlic. J Med Food.

[160] Lin GH, Lee YJ, Choi DY, Han SB, Jung JK, Hwang BY, Moon DC, Kim Y, Lee MK, Oh KW, Jeong HS, Leem JY, Shin HK, Lee JH, Hong JT (2012). Anti-amyloidogenic effect of thiacremonone through anti-inflamation in vitro and in vivo models. J Alzheimers Dis.

[161] Arunkumar R, Sharmila G, Elumalai P, Senthilkumar K, Banudevi S, Gunadharini DN, Benson CS, Daisy P, Arunakaran J (2012). Effect of diallyl disulfide on insulin-like growth factor signaling molecules involved in cell survival and proliferation of human prostate cancer cells in vitro and in silico approach through docking analysis. Phytomedicine.

[162] Shin JH, Ryu JH, Kang MJ, Hwang CR, Han J, Kang D (2013). Short-term heating reduces the anti-inflammatory effects of fresh raw garlic extracts on the LPS-induced production of NO and pro-inflammatory cytokines by downregulating allicin activity in RAW 2647 macrophages. Food Chem Toxicol..

[163] Lin X, Yu S, Chen Y, Wu J, Zhao J, Zhao Y (2012). Neuroprotective effects of diallyl sulfide against transient focal cerebral ischemia via anti-apoptosis in rats. Neurol Res.

[164] Ashafaq M, Khan MM, Shadab Raza S, Ahmad A, Khuwaja G, Javed H, Khan A, Islam F, Siddiqui MS, Safhi MM (2012). S-allyl cysteine mitigates oxidative damage and improves neurologic deficit in a rat model of focal cerebral ischemia. Nutr Res.

[165] Rojas P, Serrano-Garcia N, Medina-Campos ON, Pedraza-Chaverri J, Maldonado PD, Ruiz-Sanchez E (2011). S-Allylcysteine, a garlic compound, protects against oxidative stress in 1-methyl-4-phenylpyridinium-induced parkinsonism in mice. J Nutr Biochem.

[166] Nishiyama N, Moriguchi T, Morihara N, Saito H (2001). Ameliorative effect of S-allylcysteine, a major thioallyl constituent in aged garlic extract, on learning deficits in senescence-accelerated mice. J Nutr.

[167] Chauhan NB, Sandoval J (2007). Amelioration of early cognitive deficits by aged garlic extract in Alzheimer’s transgenic mice. Phytother Res.

[168] Jeong JH, Jeong HR, Jo YN, Kim HJ, Shin JH, Heo HJ (2013). Ameliorating effects of aged garlic extracts against Abeta-induced neurotoxicity and cognitive impairment. BMC Complement Altern Med.

[169] Bhattacharya A, Ghosal S, Bhattacharya SK (2001). Anti-oxidant effect of Withania somnifera glycol withanolides in chronic foot shock stress-induced perturbations of oxidative free radical scavenging enzymes and lipid peroxidation in rat frontal cortex and striatum. J Ethnopharmacol.

[170] Kulkarni SK, George B, Mathur R (1998). Neuroprotection by Withania somnifera root extract against lithium-pilocarpine-induced seizures. Indian Drugs.

[171] Elsakka M, Grigorescu E, Stanescu U, Stanescu U, Dorneanu V (1990). New data referring to chemistry of Withania somnifera species. Rev Med Chir Soc Med Nat Iasi Apr-Jun.

[172] Thakur RS, Puri HS, Hussain A. Major medicinal plants of India. CIMAP, Lucknow (India) (a monograph on Withania somnifera); 1987. https://www.wikidoc.org/index.php/Ashwagandha.

[173] Puri HS. Simple Ayurvedic Remedies. UBSPD, Delhi (India) (use of ashwagandha in various recipes); 2002.

[174] Narinderpal K, Junaid N, Raman B (2013). A review on pharmacological profile of Withania somnifera (Ashwagandha). Res Rev.

[175] Prabu PC, Panchapakesan S (2015). Prenatal developmental toxicity evaluation of Withania somnifera root extract in Wistar rats. Drug Chem Toxicol.

[176] Prabu PC, Panchapakesan S, Raj CD (2013). Acute and sub-acute oral toxicity assessment of the hydroalcoholic extract of Withania somnifera roots in Wistar rats. Phytother Res.

[177] Kuboyama T, Tohda C, Zhao J, Nakamura N, Hattori M, Komatsu K (2002). Axon- or dendrite-predominant outgrowth induced by constituents from Ashwagandha. NeuroReport.

[178] Tohda C, Joyashiki E (2009). Sominone enhances neurite outgrowth and spatial memory mediated by the neurotrophic factor receptor RET.. Br J Pharmacol..

[179] Kuboyama T, Tohda C, Komatsu K (2005). Neuritic regeneration and synaptic reconstruction induced by withanolide A. Br J Pharmacol.

[180] Konar A, Shah N, Singh R, Saxena N, Kaul SC, Wadhwa R, Thakur MK. Protective role of ashwagandha leaf extract and its component withanone on scopolamine-induced changes in the brain and brain-derived cells. PLoS ONE. 2011;6(11): 27265. doi*: *10.1371/journal.pone.0027265. (Epub 2011 Nov 11) 10.1371/journal.pone.0027265 PMC3214041 22096544

[181] Kumar S, Harris RJ, Seal CJ, Okello EJ (2012). An aqueous extract of withania somnifera root inhibits amyloid beta fibril formation in vitro. Phytother Res.

[182] Jayaprakasam B, Padmanabhan K, Nair MG (2010). Withanamides in withania somnifera fruit protect PC-12 cells from beta-amyloid responsible for Alzheimer’s disease. Phytother Res.

[183] Sehgal N, Gupta A, Valli RK, Joshi SD, Mills JT, Hamel E, Khanna P, Jain SC, Thakur SS, Ravindranath V (2012). Withania somnifera reverses Alzheimer’s disease pathology by enhancing low-density lipoprotein receptor-related protein in liver. Proc Natl Acad Sci USA.

[184] Dhuley JN (1998). Effect of ashwagandha on lipid peroxidation in stress-induced animals. J Ethnopharmacol.

[185] Panda S, Kar A (1997). Evidence for free radical scavenging activity of Ashwagandha root powder in mice. Indian J Physiol Pharmacol.

[186] Gupta M, Kaur G (2016). Aqueous extract from the *Withania somnifera* leaves as a potential anti-neuroinflammatory agent: a mechanistic study. J Neuroinflammation.

[187] Warrier PK, Nambiar VPK, Ramankutty C. Indian Medicinal Plants. Madras: Orient Longman Ltd; 1993. *https://doi.org/*10.1111/j.2042-7158.1994.tb05722.x

[188] Russo A, Borrelli F (2005). Bacopa monniera, a reputed nootropic plant: an overview. Phytomedicine.

[189] Chaudhari KS, Tiwari NR, Tiwari RR, Sharma RS (2017). Neurocognitive effect of nootropic drug Brahmi (Bacopa monnieri) in Alzheimer's disease. Ann Neurosci.

[190] Ramasamy S, Chin SP, Sukumaran SD, Buckle MJC, Kiew LV, Chung LY (2015). In silico and in vitro analysis of bacoside A aglycones and its derivatives as the constituents responsible for the cognitive effects of Bacopa monnieri. PLoS ONE.

[191] Vishwakarma RK, Patel K, Sonawane P, Kumari U, Singh S, Abbassi S, Agrawal DC, Tsay H-S, Khan BM (2015). Squalene synthase gene from medicinal herb Bacopa monniera: molecular characterization, differential expression, comparative modeling, and docking studies. Plant Mol Biol Rep.

[192] Uabundit N, Wattanathorn J, Mucimapura S, Ingkaninan K (2010). Cognitive enhancement and neuroprotective effects of Bacopa monnieri in Alzheimer's disease model. J Ethnopharmacol..

[193] Dwivedi S, Nagarajan R, Hanif K, Siddiqui HH, Nath C, Shukla R (2013). Standardized extract of Bacopa monniera attenuates okadaic acid induced memory dysfunction in rats: effect on Nrf2 pathway. Evid Based Complement Alternat Med..

[194] Saini N, Oelhafen S, Hua H, Georgiev O, Schaffner W, Büeler H (2010). Extended lifespan of Drosophila parkin mutants through sequestration of redox-active metals and enhancement of anti-oxidative pathways. Neurobiol Dis.

[195] Babita S, Shivani P, Satyndra KY, Rajesh V, Surya PS, Abbas AM (2017). Role of ethanolic extract of Bacopa monnieri against 1-methyl-4-phenyl-1,2,3,6-tetrahydropyridine (MPTP) induced mice model via inhibition of apoptotic pathways of dopaminergic neurons. Brain Res Bull.

[196] Channa S, Dar A, Anjum S, Yaqoob M, Atta U-R (2006). Anti-inflammatory activity of Bacopa monniera in rodents. J Ethnopharmacol.

[197] Sumathi T, Nongbri A (2008). Hepatoprotective effect of Bacoside-A, a major constituent of Bacopa monniera Linn. Phytomedicine.

[198] Rastogi M, Ojha RP, Devi BP, Aggarwal A, Agrawal A, Dubey GP (2012). Amelioration of age associated neuroinflammation on long term bacosides treatment. Neurochem Res..

[199] Barelli S, Canellini G, Thadikkaran L (2008). Oxidation of proteins: basic principles and perspectives for blood proteomics. Proteomics Clin Appl.

[200] Shinomol GK, Bharath MM (2012). Neuromodulatory propensity of Bacopa monnieri leaf extract against 3-nitropropionic acid-induced oxidative stress: in vitro and in vivo evidences. Neurotox Res..

[201] Simpson T, Pase M, Stough C (2015). Bacopa monnieri as an antioxidant therapy to reduce oxidative stress in the aging brain. Evid Based Complement Alternat Med.

[202] Maritim AC, Sanders RA, Watkins JB (2003). Diabetes, oxidative stress, and antioxidants: a review. J Biochem Mol Toxicol.

[203] Nannepaga JS, Korivi M, Tirumanyam M, Bommavaram M, Kuo CH (2014). Neuroprotective effects of Bacopa monniera whole-plant extract against aluminum induced hippocampus damage in rats: evidence from electron microscopic images. Chin J Physiol..

[204] Liu X, Yue R, Zhang J, Shan L, Wang R, Zhang W (2013). Neuroprotective effects of bacopaside I in ischemic brain injury. Restor Neurol Neurosci.

[205] Kapoor R, Saurabh S, Poonam K (2009). Bacopa monnieri modulates antioxidant responses in brain and kidney of diabetic rats. Environ Toxicol Pharmacol.

[206] Agarwal S, Chaudhary B, Bist R (2016). Bacoside A and bromelain relieve dichlorvos induced changes in oxidative responses in mice serum. Chem Biol Interact.

[207] Priyanka HP, Singh RV, Mishra M, ThyagaRajan S (2013). Diverse age-related effects of Bacopa monnieri and donepezil in vitro on cytokine production, antioxidant enzyme activities, and intracellular targets in splenocytes of F344 male rats. Int Immuno pharmacol.

[208] Pandey SP, Singh HK, Prasad S (2015). Alterations in hippocampal oxidative stress, expression of AMPA receptor GluR2 subunit and associated spatial memory loss by Bacopa monnieri extract (CDRI-08) in streptozotocin-induced diabetes mellitus type 2 mice. PLoS ONE..

[209] Das TK, Hamid MRA, Das T, Shad KF (2015). Potential of Glyco-withanolides from Withania Somnifera (Ashwagandha) as Therapeutic Agents for the Treatment of Alzheimer's Disease. World J Pharm Res.

[210] Malishev R, Shaham-Niv S, Nandi S, Kolusheva S, Gazit E, Jelinek R (2017). Bacoside-A, an Indian traditional-medicine substance, inhibits β-amyloid cytotoxicity, fibrillation, and membrane interactions. ACS Chem Neurosci.

[211] Ternchoocheep K, Ingkaninan K, Yasothornsrikul S (2012). Tau protein attenuation ability of Bacopa monnieri extract on nerve growth factor-deprived PC12 cells in normal-serum and serum-free medium. Chiang Mai Med J.

[212] Ransohoff RM (2016). A polarizing question: do M1 and M2 microglia exist?. Nat Neurosci.

[213] Viji V, Helen A (2011). Inhibition of pro-inflammatory mediators: role of *Bacopa monniera* (L.) Wettst. Inflammo Pharmacol.

[214] Hans O, David HB. 1 – Introduction to mechanisms of allergic disease. In: Saunders WB, Edinburgheds. Allergy. 2012; 2012:1–32.

[215] Cho S, Hwang ES (2011). Fluorescence-based detection and quantification of features of cellular senescence. Methods Cell Biol.

[216] Hong J, Bose M, Ju J (2004). Modulation of arachidonic acid metabolism by curcumin and related beta-diketone derivatives: effects on cytosolic phospholipase A(2), cyclooxygenases and 5-lipoxygenase. Carcinogenesis.

[217] Pianpumepong P, Anal AK, Doungchawee G, Noomhorm A (2012). Study on enhanced absorption of phenolic compounds of Lactobacillus-fermented turmeric (Curcuma longa Linn.) beverages in rats. Int J Food Sci Technol..

[218] Garcia-Alloza M, Borrelli LA, Rozkalne A, Hyman BT, Bacskai BJ (2007). Curcumin labels amyloid pathology in vivo, disrupts existing plaques, and partially restores distorted neurites in an Alzheimer mouse model. J Neurochem.

[219] Mohorko N, Repovs G, Popovic M, Kovacs GG, Bresjanac M (2010). Curcumin labeling of neuronal fibrillar tau inclusions in human brain samples. J Neuropathol Exp Neurol.

[220] Hafner-Bratkovic I, Gaspersic J, Smid LM, Bresjanac M, Jerala R (2008). Curcumin binds to the alpha-helical intermediate and to the amyloid form of prion protein—A new mechanism for the inhibition of PrP(Sc) accumulation. J Neurochem.

[221] Mosley RL, Benner EJ, Kadiu I, Thomas M, Boska MD, Hasan K, Laurie C, Gendelman HE (2006). Neuroinflammation, Oxidative Stress and the Pathogenesis of Parkinson’s Disease. Clin Neurosci Res.

[222] Giri RK, Rajagopal V, Kalra VK (2004). Curcumin, the active constituent of turmeric, inhibits amyloid peptide-induced cytochemokine gene expression and CCR5-mediated chemotaxis of THP-1 monocytes by modulating early growth response-1 transcription factor. J Neurochem.

[223] Pendurthi UR, Rao LV (2000). Suppression of transcription factor Egr-1 by curcumin. Thromb Res.

[224] Park SY, Kim DS (2002). Discovery of natural products from Curcuma longa that protect cells from beta-amyloid insult: A drug discovery effort against Alzheimers disease. J Nat Prod.

[225] Biswas SK, McClure D, Jimenez LA, Megson IL, Rahman I (2005). Curcumin induces glutathione biosynthesis and inhibits NF-kappaB activation and interleukin-8 release in alveolar epithelial cells: mechanism of free radical scavenging activity. Antioxid Redox Signal.

[226] Cho JW, Lee KS, Kim CW (2007). Curcumin attenuates the expression of IL-1beta, IL-6, and TNF-alpha as well as cyclin E in TNF-alpha-treated HaCaT cells; NF-kappaB and MAPKs as potential upstream targets. Int J Mol Med.

[227] Gulcubuk A, Altunatmaz K, Sonmez K, Haktanir-Yatkin D, Uzun H, Gurel A, Aydin S (2006). Effects of curcumin on tumour necrosis factor-alpha and interleukin-6 in the late phase of experimental acute pancreatitis. J Vet Med A Physiol Pathol Clin Med..

[228] Jat D, Parihar P, Kothari SC, Parihar MS (2013). Curcumin reduces oxidative damage by increasing reduced glutathione and preventing membrane permeability transition in isolated brain mitochondria. Cell Mol Biol..

[229] Jones DP (2008). Radical-free biology of oxidative stress. Am J Physiol Cell Physiol.

[230] Wanninger S, Lorenz V, Subhan A, Edelmann FT (2015). Metal complexes of curcumin—Synthetic strategies, structures and medicinal applications. Chem Soc Rev.

[231] Mishra S, Palanivelu K (2008). The effect of curcumin (turmeric) on Alzheimer’s disease: an overview. Ann Indian Acad Neurol.

[232] Shakibaei M, John T, Schulze-Tanzil G, Lehmann I, Mobasheri A (2007). Suppression of NF-kappaB activation by curcumin leads to inhibition of expression of cyclo-oxygenase-2 and matrix metalloproteinase-9 in human articular chondrocytes: Implications for the treatment of osteoarthritis. Biochem Pharmacol.

[233] Kozmon S, Tvaroška I (2015). Molecular dynamic studies of amyloid-beta interactions with curcumin and Cu2+ ions. Chem Papers.

[234] Soni K, Kuttan R (1992). Effect of oral curcumin administration on serum peroxides and cholesterol levels in human volunteers. Indian J Physiol Pharmacol.

[235] Lim GP, Chu T, Yang F (2001). Cole GM The curry spice curcumin reduces oxidative damage and amyloid pathogenesis on Alzheimer's transgenic mouse. J Neurosci.

[236] Kim GY, Kim KH, Lee SH, Yoon MS, Lee HJ, Moon DO (2005). Curcumin inhibits immunostimulatory function of dendritic cells: MAPKs and translocation of NF-B as potential targets. J Immunol.

[237] Kim KH, Lee D, Lee HL, Kim C-E, Jung K, Kang KS (2017). Beneficial effects ofPanax ginseng for the treatment and prevention of neurodegenerative diseases: past findings and futuredirections. J Ginseng Res.

[238] Lu G-H, Zhou Q, Sun S-Q, Leung KS-Y, Zhang H, Zhao Z-Z (2008). Differentiation of Asian ginseng, American ginseng and Not ginseng by Fourier transform infrared spectroscopy combined with two-dimensional correlation infrared spectroscopy. J Mol Struct.

[239] Lu G-H, Zhou Q, Sun S-Q, Leung KS-Y, Zhang H, Zhao Z-Z (2008). Differentiation of Asian ginseng, American ginseng and Notoginseng by Fourier transform infrared spectroscopy combined with two-dimensional correlation infrared spectroscopy. J Mol Struct.

[240] Christensen LP. Chapter 1 ginsenosides. Chemistry, biosynthesis, analysis, and potential health effects. Adv Food Nutr Res 2018; 55: 1–99. 10.1016/S1043-4526(08)00401-4 18772102

[241] Vassar R, Bennett BD, Babu-Khan S, Kahn S, Mendiaz EA, Denis P, Teplow DB, Ross S, Amarante P, Loeloff R (1999). Beta-secretase cleavage of Alzheimer's amyloid precursor protein by the transmembrane aspartic protease BACE. Science.

[242] Li L, Liu J, Yan X (2011). Protective effects of ginsenoside Rdagainst okadaic acid-induced neurotoxicity in vivo and in vitro. J Ethnopharmacol.

[243] Ikonomovic MD, Mufson EJ, Wuu J, Bennett DA, DeKosky ST (2005). Reduction of choline acetyltransferase activity in primary visual cortex in mild to moderate Alzheimer's disease. Arch Neurol.

[244] Choi RJ, Roy A, Jung HJ, Ali MY, Min BS, Park CH, Yokozawa T, Fan TP, Choi JS, Jung HA (2016). BACE1 molecular docking and anti-Alzheimer's disease activities of ginsenosides. J Ethnopharmacol.

[245] Kim SF, Huri DA, Snyder SH (2005). Inducible nitric oxide synthase binds, S-nitrosylates, and activates cyclooxygenase-2. Science.

[246] Wang Y, Liu J, Zhang Z, Bi P, Qi Z, Zhang C (2011). Anti neuroinflammation effect of ginsenoside Rbl in a rat model of Alzheimer disease. Neurosci Let.

[247] Muhammad I, Rahat U, Amjad K, Myeong OK (2020). Ongoing research on the role of gintonin in the management of neurodegenerative disorders. Cells.

[248] Caspersen CJ, Powell KE, Christenson GM (1985). Physical activity, exercise, and physical fitness: definitions and distinctions for health-related research. Public Health Rep.

[249] Vina J, Sanchis-Gomar F, Martinez-Bello V, Gomez-Cabrera MC (2012). Exercise acts as a drug; the pharmacological benefits of exercise. Br J Pharmacol.

[250] Voss MW, Vivar C, Kramer AF, van Praag H (2013). Bridging animal and human models of exercise-induced brain plasticity. Trends Cogn Sci..

[251] Colcombe S, Kramer AF (2003). Fitness effects on the cognitive function of older adults: a meta-analytic study. Psychol Sci.

[252] Meeusen R, De Meirleir K (2015). Exercise and brain neurotransmission. Sports Med.

[253] Gomez-Pinilla F, Dao L, So V (1997). Physical exercise induces FGF-2 and its mRNA in the hippocampus. Brain Res.

[254] Ding Q, Vaynman S, Akhavan M, Ying Z, Gomez-Pinilla F (2006). Insulin-like growth factor I interfaces with brain-derived neurotrophic factor-mediated synaptic plasticity to modulate aspects of exercise-induced cognitive function. Neuroscience.

[255] Uysal N, Kiray M, Sisman AR, Camsari UM, Gencoglu C, Baykara B, Cetinkaya C, Aksu I (2015). Effects of voluntary and involuntary exercise on cognitive functions, and VEGF and BDNF levels in adolescent rats. Biotech Histochem.

[256] Vaynman S, Gomez-Pinilla F (2005). License to run: exercise impacts functional plasticity in the intact and injured central nervous system by using neurotrophins. Neurorehabil Neural Repair.

[257] Radak Z, Toldy A, Szabo Z, Siamilis S, Nyakas C, Silye G, Jakus J, Goto S (2006). The effects of training and detraining on memory, neurotrophins and oxidative stress markers in rat brain. Neurochem Int.

[258] Kim DH, Ko IG, Kim BK, Kim TW, Kim SE, Shin MS, Kim CJ, Kim H, Kim KM, Baek SS (2010). Treadmill exercise inhibits traumatic brain injury-induced hippocampal apoptosis. Physiol Behav.

[259] Brown BM, Peiffer JJ, Martins RN (2013). Multiple effects of physical activity on molecular and cognitive signs of brain aging: can exercise slow neurodegeneration and delay Alzheimer’s disease?. Mol Psychiatry.

[260] Rovio S, Kareholt I, Helkala EL, Viitanen M, Winblad B, Tuomilehto J, Soininen H, Nissinen A, Kivipelto M (2005). Leisure-time physical activity at midlife and the risk of dementia and Alzheimer’s disease. Lancet Neurol.

[261] Masters CL, Beyreuther K (1995). Molecular neuropathology of Alzheimer’s disease. Arzneimittelforschung.

[262] Thompson PM, Hayashi KM, Dutton RA, Chiang MC, Leow AD, Sowell ER, DeZubicaray G, Becker JT, Lopez OL, Aizenstein HJ, Toga AW (2007). Tracking Alzheimer’s disease. Ann N Y Acad Sci.

[263] Ikonomovic MD, Abrahamson EE, Price JC, Hamilton RL, Mathis CA, Paljug WR, Debnath ML, Cohen AD, Mizukami K, DeKosky ST, Lopez OL (2008). Post-mortem correlates of in vivo PiB-PET amyloid imaging in a typical case of Alzheimer’s disease. Brain.

[264] Um HS, Kang EB, Leem YH, Cho IH, Yang CH, Chae KR, Hwang DY, Cho JY (2008). Exercise training acts as a therapeutic strategy for reduction of the pathogenic phenotypes for Alzheimer’s disease in an NSE/APPsw-transgenic model. Int J Mol Med.

[265] Gratuze M, Julien J, Morin F, Marette A, Planel E (2017). Differential effects of voluntary treadmill exercise and caloric restriction on tau pathogenesis in a mouse model of Alzheimer’s disease-like tau pathology fed with Western diet. Prog Neuropsychopharmacol Biol Psychiatry.

[266] Jeong JH, Kang EB (2018). Effects of treadmill exercise on PI3K/AKT/GSK-3beta pathway and tau protein in high-fat diet-fed rats. J Exerc Nutrition Biochem..

[267] Kang EB, Cho JY (2015). Effect of treadmill exercise on PI3K/AKT/mTOR, autophagy, and Tau hyperphosphorylation in the cerebral cortex of NSE/htau23 transgenic mice. J Exerc Nutrition Biochem.

[268] Leem YH, Lim HJ, Shim SB, Cho JY, Kim BS, Han PL (2009). Repression of tau hyperphosphorylation by chronic endurance exercise in aged transgenic mouse model of tauopathies. J Neurosci Res.

[269] Ohia-Nwoko O, Montazari S, Lau YS, Eriksen JL (2014). Long-term treadmill exercise attenuates tau pathology in P301S tau transgenic mice. Mol Neurodegener.

[270] Belarbi K, Burnouf S, Fernandez-Gomez FJ, Laurent C, Lestavel S, Figeac M, Sultan A, Troquier L, Leboucher A, Caillierez R, Grosjean ME, Demeyer D, Obriot H, Brion I, Barbot B, Galas MC, Staels B, Humez S, Sergeant N, Schraen-Maschke S, Muhr-Tailleux A, Hamdane M, Buee L, Blum D (2011). Beneficial effects of exercise in a transgenic mouse model of Alzheimer’s disease-like Tau pathology. Neurobiol Dis.

[271] Elahi M, Motoi Y, Matsumoto SE, Hasan Z, Ishiguro K, Hattori N (2016). Short-term treadmill exercise increased tau insolubility and neuroinflammation in tauopathy model mice. Neurosci Lett.

[272] Nichol KE, Poon WW, Parachikova AI, Cribbs DH, Glabe CG, Cotman CW (2008). Exercise alters the immune profile in Tg2576 Alzheimer mice toward a response coincident with improved cognitive performance and decreased amyloid. J Neuroinflam.

[273] Parachikova A, Nichol KE, Cotman CW (2008). Short-term exercise in aged Tg2576 mice alter neuroinflammation and improves cognition. Neurobiol Dis.

[274] Liu HL, Zhao G, Zhang H, Shi LD (2013). Long-term treadmill exercise inhibits the progression of Alzheimer’s disease-like neuropathology in the hippocampus of APP/PS1 transgenic mice. Behav Brain Res.

[275] Baker LD, Bayer-Carter JL, Skinner J, Montine TJ, Cholerton BA, Callaghan M, Leverenz JB, Walter BK, Tsai E, Postupna N, Lampe J, Craft S (2012). High intensity physical activity modulates diet effects on cerebrospinal amyloid-beta levels in normal aging and mild cognitive impairment. J Alzheimer’s Dis.

[276] Law LL, Rol RN, Schultz SA, Dougherty RJ, Edwards DF, Koscik RL, Gallagher CL, Carlsson CM, Bendlin BB, Zetterberg H, Blennow KC (2018). Moderate intensity physical activity associates with CSF biomarkers in a cohort at risk for Alzheimer’s disease. Alzheimer’s Dement.

[277] Brown BM, Rainey-Smith SR, Dore V, Peiffer JJ, Burnham SC, Laws SM, Taddei K, Ames D, Masters CL, Rowe CC, Martins RN, Villemagne VL (2018). Self-reported physical activity is associated with tau burden measured by positron emission tomography. J Alzheimer’s Dis.

[278] Goto S, Naito H, Kaneko T, Chung HY (2007). Hormetic effects of regular exercise in aging: correlation with oxidative stress. Appl Physiol Nutr Metab.

[279] Devi SA, Kiran TR (2004). Regional responses in antioxidant system to exercise training and dietary vitamin E in aging rat brain. Neurobiol Aging.

[280] Hyun S, Lee JH, Jin H, Nam J, Namkoong B, Lee G, Chung J, Kim VN (2009). Conserved MicroRNA miR-8/miR-200 and Its Target USH/FOG2 Control Growth by Regulating PI3K. Cell.

[281] Kaytor MD, Orr HT (2002). The GSK3 beta signaling cascade and neurodegenerative disease. Curr Opin Neurobiol.

